# The link of carbon catabolite repression elements, small RNAs CrcY and CrcZ and polyhydroxyalkanoate metabolism in *Pseudomonas putida* KT2440

**DOI:** 10.1186/s13068-025-02707-5

**Published:** 2025-10-17

**Authors:** Yixin Che, Dominic Harris-Jukes, Elizabeth Sitko, Moya Brady, William Casey, Michael P. Shaver, Kevin O’Connor, Tanja Narancic

**Affiliations:** 1 UCD Earth Institute and School of Biomolecular and Biomedical Science, University College Dublin, Belfield, Dublin 4, Ireland; 2 BiOrbic—Bioeconomy Research Centre, University College Dublin, Belfield, Dublin 4, Ireland; 3 Sustainable Materials Innovation Hub, Department of Materials, University of Manchester, Oxford Road, Manchester, M13 9PL UK; 4 O’Brien Centre for Science, Bioplastech LTD (BIOPLASTECH), University College of Dublin, Dublin, Ireland

## Abstract

**Background:**

Polyhydroxyalkanoates (PHAs), biodegradable polymers, can be synthesised and degraded by a number of bacteria. With a range of monomer composition and molecular weight, these polymers can be used for packaging to medical applications. However, the production cost, inadequate mechanical properties, and challenging melt processing properties are major impediments.

Understanding and harnessing the regulatory networks underpinning PHA production in a model organism *Pseudomonas putida* KT2440 is an invaluable tool to increase PHA production and alter polymer properties for specific applications.

**Results:**

The small RNAs CrcY and CrcZ, key components of the carbon catabolite repression (CCR) system, are implicated in PHA metabolism in *P. putida* KT2440. Their *in trans* overexpression in *P. putida* KT2440 shows a 1.3- to 3.5-fold increase in PHA titre (g/L), using glucose or octanoate as feedstocks. This is accompanied by a decrease in the Mw of the synthesised polymer. Among the proteins showing differential expression in response to CrcY and CrcZ overexpression, glutaryl-CoA dehydrogenase GcdH, involved in the catabolism of lysine, hydroxylysine, and tryptophan, and gamma-glutamyl transpeptidase GGT, involved in glutathione metabolism, showed a consistent increase in abundance across different conditions. It also appears that CrcY and CrcZ can compensate for each other, as only when both sRNAs are removed is a 2.5-fold decrease in PHA observed. We also show that these sRNAs require other CCR elements, Hfq and Crc, for their role in PHA metabolism.

**Conclusions:**

One strategy to overcome poor mechanical properties of PHAs is to blend them with a second polymer. Medium chain length (mcl)-PHA acts as a plasticiser when blended with poly-3-hydroxybutyrate (PHB), the most widespread used PHA resin. Here we show a clear effect of the overexpression of CCR elements CrcY and CrcZ in *P. putida* KT2440 on the amount of the accumulated mcl-PHA and its Mw, making this tool valuable to produce mcl-PHA-based additives.

These findings highlight the complementary regulatory roles of CrcY and CrcZ in modulating CCR to optimise PHA production. This study provides insights into leveraging CCR elements to enhance the efficiency of PHA biosynthesis, contributing to the development of sustainable bioplastic production.

**Supplementary Information:**

The online version contains supplementary material available at 10.1186/s13068-025-02707-5.

## Introduction

Polyhydroxyalkanoates (PHAs) are a family of biopolymers that can be synthesised and degraded by various bacteria [1]. They are polymers of (*R*)-3-hydroxyalkanoic acids connected by energetic ester bonds [2]. More than 150 different monomers of PHAs have been reported [3]. Depending on the type of monomer constituents, PHAs can be classified into short-chain-length PHA consisting of monomers of four or five carbon atoms (scl-PHA, C4 and C5) and medium-chain-length PHA (mcl-PHA, C6 to C14) [4]. The diverse monomer composition and wide range of molecular weights (5 × 10^4^ to 2 × 10^7^ Da) give PHAs a broad spectrum of thermal and mechanical properties [2]. This versatility has led to suggested applications for PHAs, including agricultural mulch films [5], plastic packaging [6], and in biomedical applications for drug delivery and tissue engineering [7]. However, although PHAs are touted as a replacement for conventional polymers such as polypropylene (PP) or polyethylene (PE), their market presence is minuscule [8]. The polymers, while diverse, show limited thermal stability, granting them a very narrow processing window, and challenging mechanical properties including brittleness for scl-PHA or low strength for mcl-PHA, and low melting temperature [9].

PHAs are synthesised in bacteria under specific conditions, usually a nutrient imbalance with excess carbon present and a limited concentration of an inorganic nutrient, and many regulatory levels and elements are involved. Understanding the interplay of these regulatory elements and harnessing them could provide us with a means to better control the monomer composition and molecular weight of these polymers, and thereby better control their properties.

**Fig. 1:**
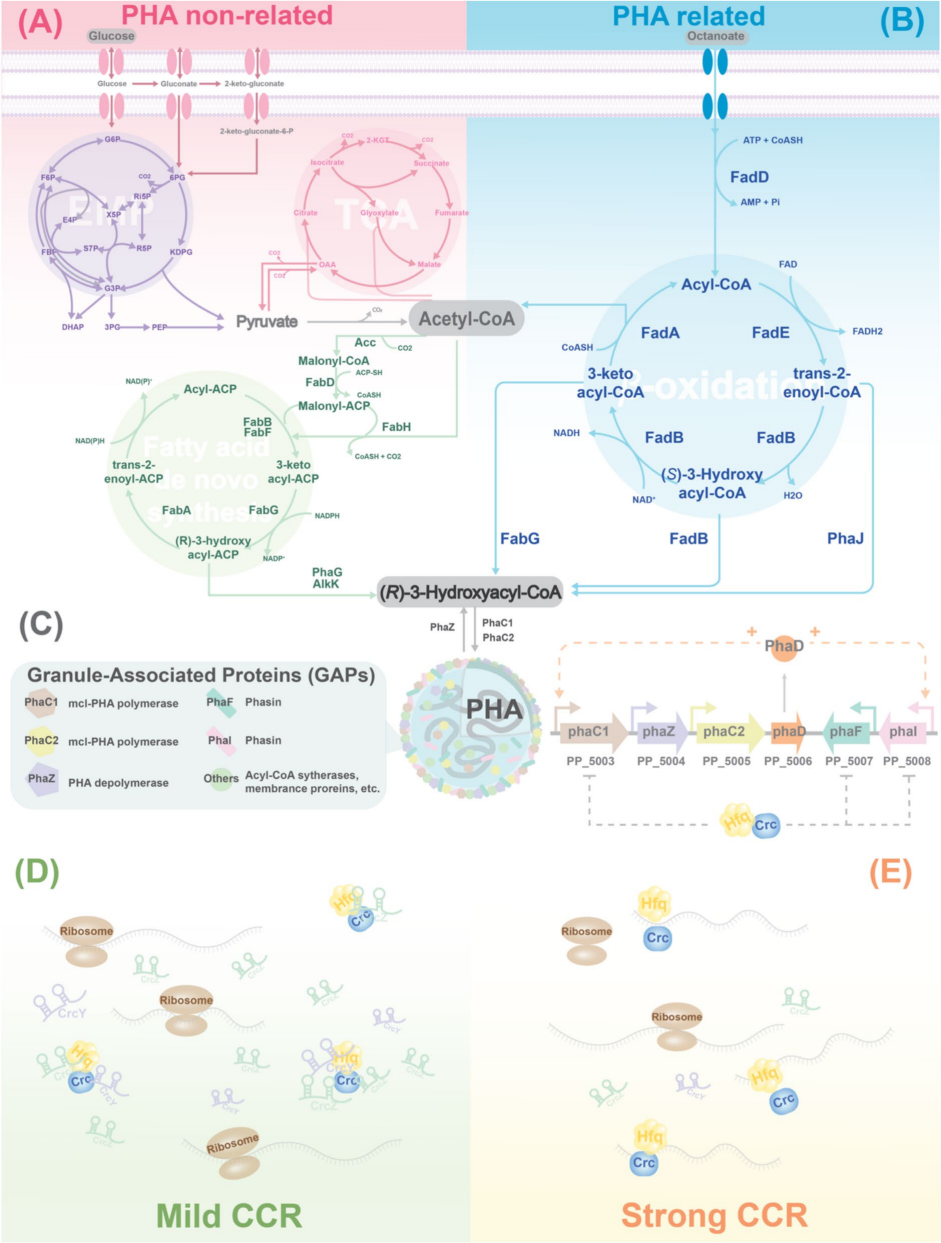
Integrated overview of PHA metabolism, PHA gene cluster organisation, and CCR regulation in *Pseudomonas putida* KT2440. **A** De novo fatty acid synthesis used as a main PHA-monomer supplier pathway when carbon-unrelated substrates are used. **B** The link of *β*-oxidation and PHA synthesis when carbon-related substrates are used. **C** The PHA granule with key phasins and *pha* gene cluster. CCR regulatory mechanism under mild (**D**) and strong (**E**) repression conditions

*Pseudomonas putida* KT2440 is one of the most studied model organisms for mcl-PHA synthesis [10]. It can use a wide range of carbon sources to synthesise PHA at a high carbon to nitrogen or carbon-to-phosphate ratio [11, 12]. Two main pathways are used for PHA synthesis by KT2440, as shown in Fig. 1A, B. When fatty acids, also known as PHA monomer-related carbon sources, are used, they are oxidised via the *β*-oxidation pathway [11]. Here, after an activated fatty acid is oxidised to *trans*-2-enoyl-CoA, this intermediate can be hydrated by one of the (*R*)-specific hydratase homologues to yield an (*R*)-3-hydroxyalkanoyl-CoA [13]. This molecule is then a direct substrate for the PHA synthases PhaC, which polymerises it into mcl-PHA. In this case, the growth and PHA accumulation are coupled processes [14]. For PHA-non-related substrates, such as glycolytic and gluconeogenic substrates, as well as aromatic compounds, the de novo fatty acid synthesis pathway is the main provider of PHA monomers. These substrates undergo central carbon metabolism to be converted into acetyl-CoA, which is then used in de novo fatty acid synthesis [15]. Here, the growth and PHA accumulation are not coupled, and a stress such as inorganic nutrient limitation is required to induce PHA accumulation [16].

PHAs accumulate as intracellular granules, the surface of which contains protein components involved in PHA metabolism, as shown in Fig. 1C. These PHA granule-associated proteins (GAPs), which include both functional and regulatory elements, are encoded by the PHA gene cluster. In *P. putida* KT2440, the cluster consists of two operons: *phaC1ZC2D* and *phaIF*, encoding the enzymes essential for PHA synthesis and degradation [15]. Within this cluster, the *phaC1* and *phaC2* genes encode two PHA synthases, *phaZ* encodes a PHA depolymerase, *phaD* encodes a TetR-like transcriptional regulator, and *phaI* and *phaF* encode PHA granule-associated phasin proteins [17]. PhaF and PhaD are involved in the transcriptional activation of the *pha* operon [18, 19].

Apart from the direct regulation of the PHA cluster by PhaD and PhaF, one of the global regulatory mechanisms linked to PHA metabolism is the carbon catabolite repression (CCR) system, commonly described in bacteria. This system optimises carbon utilisation by preferentially utilising certain substrates from a mixture of carbon sources, by repressing the expression of enzymes involved in the utilisation of less preferred carbon sources. The current CCR system model in *Pseudomonas* shows that CCR involves a regulatory cascade consisting of the two-component system (TCS) CbrA/CbrB, the proteins Hfq and Crc, and the small RNAs CrcY and CrcZ [20]. Hfq has the ability to directly bind to mRNA sequences containing the catabolite activity (CA) motif (defined as AAnAAnAA) [21, 22]. When Crc interacts with Hfq and the target mRNA, they form a complex known as the Hfq/Crc/mRNA complex [23, 24]. This complex obstructs ribosome binding to the target mRNA, thereby repressing translation. CrcY and CrcZ modulate CCR response by sequestering the Hfq/Crc complex [21, 24–26]. It appears to be a dose-dependent response, as illustrated in Fig. 1(D–E), where the more of Hfq/Crc complexes are occupied by small RNAs CrcY and CrcZ, the more of the target mRNA becomes free from CCR, alleviating the repression caused by the complex [20]. The transcriptional levels of CrcY and CrcZ impact the strength of CCR regulation, i.e. it is not an “on/off” type of regulation [25]. Their expression is controlled by the CbrA/CbrB two-component system [27], where the sensor kinase CbrA responds to environmental cues such as carbon sources and the carbon/nitrogen ratio, although the precise mechanism of CbrA activation remains unclear [28–30].

Three genes within the PHA cluster (phaC1, phaF, and phaI) contain the CA motif, suggesting potential regulation by the CCR system. Additionally, Crc has been shown to participate in the post-transcriptional regulation of the PHA synthase PhaC1 [31]. However, it is now clear that Crc lacks mRNA binding ability [32, 33]. To clarify whether CCR elements are directly or indirectly linked to PHA accumulation, a series of CCR element deletion mutants was generated, and growth and PHA accumulation were analysed using a model PHA-related substrate, fatty acid octanoate (referred to as sodium octanoate), and a model carbon-unrelated substrate, glucose. The overexpression of CrcY or CrcZ was shown to increase PHA level, and the proteome of the overexpression strains was analysed to explore the regulatory link of CCR elements and PHA metabolism in *P. putida* KT2440.

## Results

### Overexpression of *CrcY* or *CrcZ* increases PHA accumulation

**Fig. 2:**
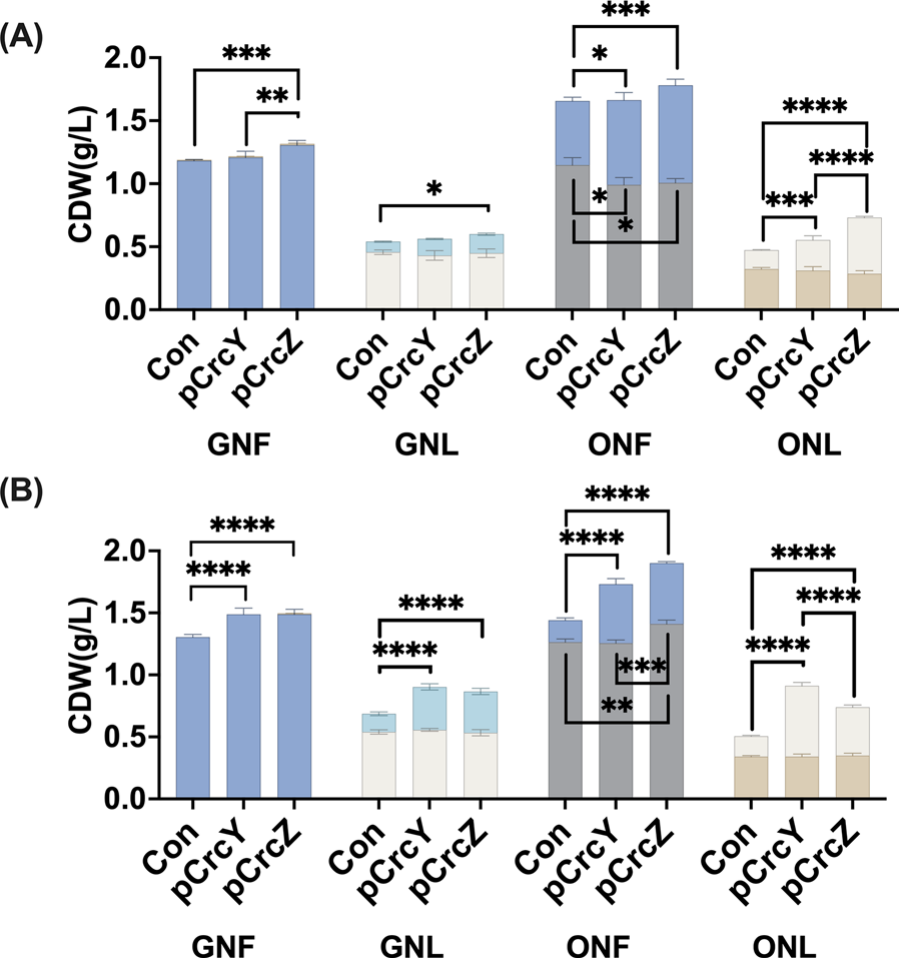
Influence of CrcY and CrcZ overexpression on biomass and PHA accumulation in *P. putida* KT2440. pBT’Tmcs vector was used for the *in trans* overexpression of CrcY (pBT'T-crcY; pCrcY), or CrcZ (pBT'T-crcZ; pCrcZ). The KT2440 strain transformed with an empty pBT'T was used as a control (Con). The strains were cultivated for 24 h (**A**) or 48 h (**B**) in defined medium MSM supplemented with octanoate and glucose. PHA-accumulating and non-accumulating conditions were applied: glucose (G), octanoate (O), with nitrogen conditions as nitrogen-full (NF) or nitrogen-limited (NL). PHA amounts are represented by upper bars, while biomass (excluding PHA) is depicted by bottom bars. Asterisks indicate significant differences between the samples and the control: * P ≤ 0.05, ** P ≤ 0.01, *** P ≤ 0.001, **** P ≤ 0.0001. Values are mean ± SD (n = 3 biological replicates)

CrcY and CrcZ were overexpressed in *P. putida* KT2440 *in trans* using pBT’Tmcs, allowing for constitutive expression from the P_*tac*_-promoter [34, 35]. This provides an increased level of the two sRNAs independent of native expression under the CCR control. Overexpression strains were cultivated in MSM supplemented with glucose or sodium octanoate as carbon sources. Glucose is considered a non-preferred carbon source in *Pseudomonas* species, which exhibits a reverse pattern of carbon catabolite repression (CCR) compared to the classic model in *E. coli*, where glucose is the most favoured substrate and preferentially utilised [36]. In non-PHA accumulating conditions, i.e. no inorganic nutrient limitation applied (glucose nitrogen full condition, GNF), neither the control strain nor the sRNA-overexpression strains accumulated PHA within the 48 h of incubation. It is worth mentioning that the biomass increased by 14% in the overexpression strains compared to the control (Fig. 2B). Under nitrogen limitation with glucose as a carbon and energy source (GNL), KT2440 expressing CrcY (pCrcY) or CrcZ (pCrcZ) produced over twofold more PHA compared to the control strain (Con; 0.34 g/L vs. 0.15 g/L). The non-PHA biomass showed no significant difference. This increased level of PHA accumulation in pCrcY and pCrcZ was noted after 24 h of incubation (Fig. 2A), where a 1.6- and 1.8-fold increase was observed for pCrcY and pCrcZ strains compared to Con.

Organic acids, such as succinate and acetate, are preferred carbon sources for *Pseudomonads* [37], while long or medium-chain-length fatty acids have rarely been explored in the hierarchical management of the CCR system. As mentioned, fatty acids support PHA accumulation even in the absence of nutrient limitation. Therefore, the effect of CrcY and CrcZ overexpression was investigated with sodium-octanoate, a PHA-related substrate. All strains accumulated PHA in both nitrogen-limited and nitrogen-full conditions, with pCrcY and pCrcZ strains showing a marked increase in PHA level (Fig. 2). Under nitrogen-full conditions with octanoate (ONF), PHA constituted a higher percentage of total biomass (% of cell dry weight, CDW) in the early stationary phase (24 h) compared to the late stationary phase (48 h), which contrasts with the other conditions shown in Fig. 2. The control strain accumulated 33.3% CDW (0.51 g/L) PHA, whereas pCrcY and pCrcZ produced 40.4% CDW (0.67 g/L) and 43.4% CDW (0.77 g/L) PHA (Fig. 2A), representing a 1.3-fold increase in PHA production compared to the control at 24 h. Both pCrcY and pCrcZ demonstrated a 2.7-fold higher PHA production compared to the control at 48 h (Fig. 2B). The residual biomass (excluding PHA) increased by 1.3- and 1.4-fold in pCrcY and pCrcZ, respectively. It was, however, observed that the PHA level decreased to 27.45% CDW (0.48 g/L) in pCrcY and 25.77% CDW (0.49 g/L) in pCrcZ at 48 h compared to the 24 h sample. Con also showed a decrease in both total biomass (1.48 g/L CDW at 48 h vs. 1.67 g/L CDW at 24 h) and PHA content (0.18 g/L PHA). This is likely due to carbon substrate depletion.

Under octanoate nitrogen-limited (ONL) conditions, the pCrcY strain exhibited the highest PHA level among all tested conditions at 48 h of incubation, reaching 63% of CDW (0.57 g/L), nearly doubling the control’s 33% of CDW (0.17 g/L). This corresponds to a 3.5-fold increase in PHA production compared to the control. The pCrcZ strain also showed a 2.4-fold increase in PHA production. The PHA-free biomass remained consistent across strains in ONL conditions, demonstrating that under the PHA-accumulating conditions and using single substrates, the overexpression of CrcY or CrcZ seems to have affected only the PHA level.

In addition to total PHA titres or PHA content (%CDW), the substrate-to-product conversion efficiency was analysed for both PHA and non-PHA biomass fractions (Table 1). After 48 h of incubation, the carbon substrates were fully depleted from the supernatant, as confirmed by HPLC and GC analysis. The yields were calculated based on the total amount of carbon substrate initially present in the media. While PHA production increased markedly in pCrcY and pCrcZ strains compared to the control, the efficiency of carbon source utilisation toward PHA also improved, particularly under nitrogen-limited conditions. For instance, under GNL conditions, both overexpression strains showed a 2.3-fold increase in PHA yield per gram of glucose consumed (0.07 g/g vs. 0.03 g/g). Similarly, both pCrcY and pCrcZ showed higher PHA yields under octanoate-supplemented conditions, with pCrcY reaching a maximum PHA yield of 0.17 g/g under ONL conditions, which was 3.4-fold higher than that of the control. Only marginal changes were observed in non-PHA biomass yields across conditions. These results support the conclusion that the overexpression of CrcY and CrcZ promotes carbon flux specifically toward PHA biosynthesis, without significantly altering non-PHA biomass formation.

**Table 1:** Substrate-to-product yields (g/g substrate) of PHA and non-PHA biomass in pCrcY, pCrcZ, and control strains. Under GNF and GNL conditions, yields represent grams of non-PHA or PHA produced per gram of glucose consumed; under ONF and ONL conditions, yields are calculated per gram of octanoate consumed. The pBT’Tmcs vector was used for *in trans* overexpression of CrcY (pBT'T-crcY; pCrcY) and CrcZ (pBT'T-crcZ; pCrcZ), while KT2440 harbouring the empty vector (pBT'T) served as the control. Strains were cultivated for 48 h in defined MSM medium supplemented with glucose or octanoate. PHA-accumulating and non-accumulating conditions were applied using either glucose (G) or octanoate (O) as carbon sources, under nitrogen-full (NF) or nitrogen-limited (NL) conditions

Conditions	Strains	Non-PHA yields(g/g substrate)	PHA yields (g/g substrate)
GNF	Control	0.27 ± 0.00	0.00 ± 0.00
	pCrcY	0.31 ± 0.01	0.00 ± 0.00
	pCrcZ	0.31 ± 0.01	0.00 ± 0.00
GNL	Control	0.11 ± 0.00	0.03 ± 0.00
	pCrcY	0.11 ± 0.00	0.07 ± 0.01
	pCrcZ	0.11 ± 0.01	0.07 ± 0.01
ONF	Control	0.37 ± 0.01	0.05 ± 0.01
	pCrcY	0.37 ± 0.01	0.14 ± 0.01
	pCrcZ	0.42 ± 0.01	0.15 ± 0.00
ONL	Control	0.10 ± 0.00	0.05 ± 0.00
	pCrcY	0.10 ± 0.01	0.17 ± 0.01
	pCrcZ	0.10 ± 0.01	0.12 ± 0.01

The overexpression of either of the two sRNAs, CrcY or CrcZ, had a positive effect on PHA accumulation regardless of whether the carbon source was preferred (octanoate) or non-preferred (glucose). However, the effect was more pronounced in octanoate-grown cells.

### Characterisation of PHA produced by overexpression strains

The PHA monomer composition remained unchanged in the overexpression strains compared to the control strain under the same cultivation conditions (Table 2). Additionally, no consistent differences were observed between pCrcY and pCrcZ strains in terms of PHA accumulation levels or monomer distribution. Since the pCrcY strain produced the highest PHA content at 48 h under ONL conditions, we selected the pCrcY strain for further physicochemical analysis of PHA.

**Table 2:** The PHA monomer distribution (mass/mass) and PHA accumulation level (%CDW) in pCrcY and pCrcZ under GNL, ONF, ONL conditions

PHA monomer distribution (%)		C6	C8	C10	C12	PHA (%CDW)
GNL	Control	2.47 ± 0.05	20.86 ± 0.14	72.14 ± 0.17	4.53 ± 0.02	21.31 ± 1.53
	pCrcY	2.49 ± 0.17	20.61 ± 0.09	72.51 ± 0.09	4.39 ± 0.06	38.09 ± 2.14
	pCrcZ	2.56 ± 0.10	19.60 ± 0.17	73.22 ± 0.07	4.61 ± 0.12	38.63 ± 2.88
ONF	Control	6.85 ± 0.03	87.62 ± 2.16	3.41 ± 1.06	2.12 ± 1.09	11.76 ± 0.32
	pCrcY	6.73 ± 0.23	90.28 ± 0.18	2.19 ± 0.06	0.80 ± 0.05	27.45 ± 1.53
	pCrcZ	6.82 ± 0.06	89.31 ± 0.68	2.18 ± 0.03	1.69 ± 0.70	25.77 ± 0.89
ONL	Control	8.14 ± 0.04	89.59 ± 0.18	1.43 ± 0.06	0.83 ± 0.23	32.52 ± 1.40
	pCrcY	7.15 ± 0.07	90.60 ± 0.04	1.87 ± 0.05	0.38 ± 0.07	62.51 ± 2.47
	pCrcZ	7.11 ± 0.04	90.74 ± 0.13	1.64 ± 0.05	0.51 ± 0.07	52.72 ± 0.93

**Fig. 3:**
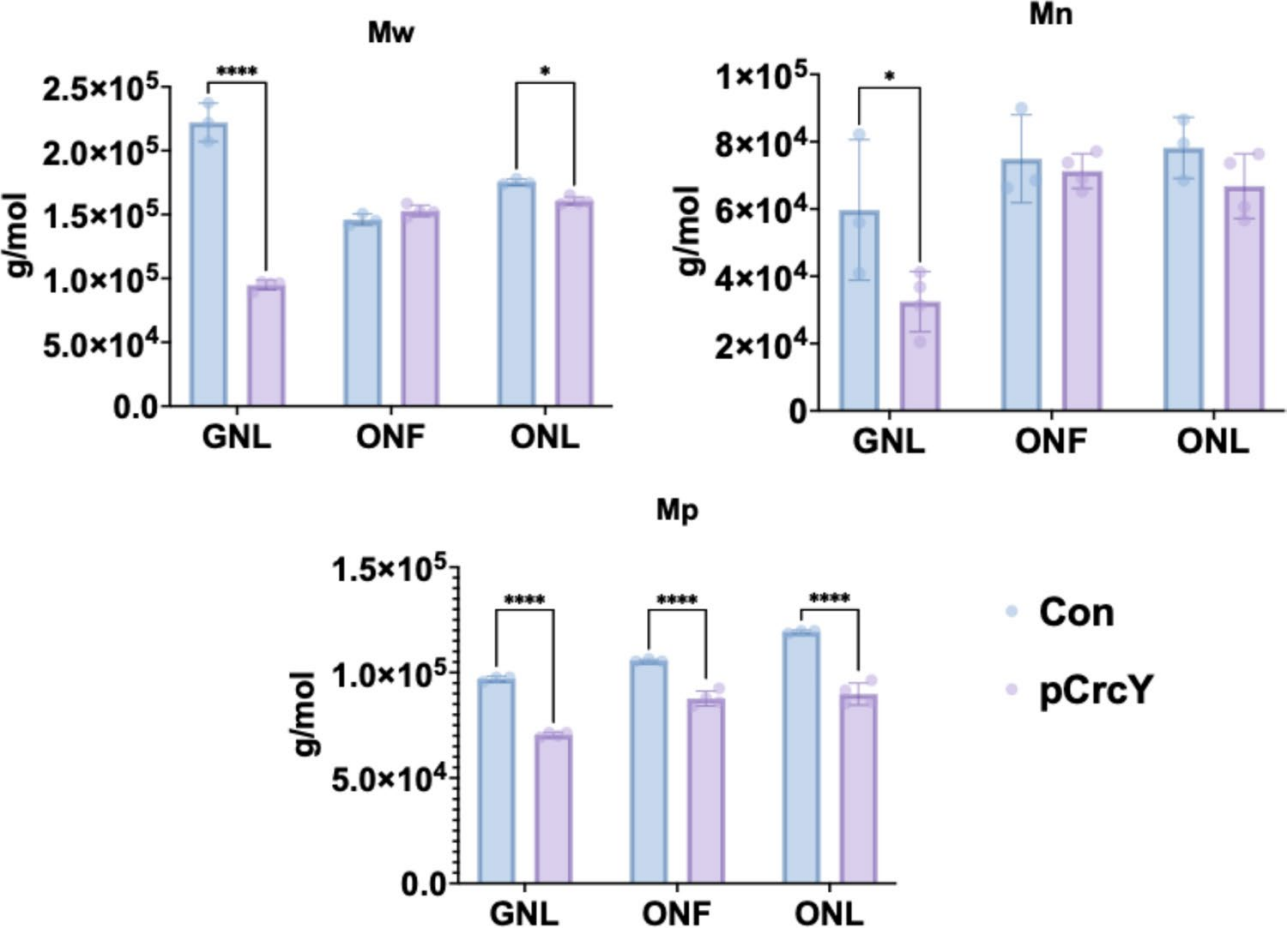
GPC analysis of mcl-PHA from pCrcY and control. The data include: Mw (weight-average molecular weight), Mn (number-average molecular weight), Mp (peak molecular weight). Asterisks indicate significant differences between the samples and the control, or the comparison indicated in brackets: * P ≤ 0.05, ** P ≤ 0.01, *** P ≤ 0.001, **** P ≤ 0.0001. Values are mean ± SD (n = 3 technical replicates)

Under GNL conditions, the control strain exhibited significantly higher molecular weights across all parameters (Fig. 3), including Mw (weight-average molecular weight), Mn (number-average molecular weight), Mp (peak molecular weight). This suggests that overexpression of CrcY negatively affects the polymer chain length, resulting in lower molecular weights. Specifically, the peak molecular weight (Mp) of the control was 9.705 × 10^4^ g/mol, compared to 7.058 × 10^4^ g/mol for pCrcY, representing a 27.3% reduction in the overexpression strain. Similarly, the number-average molecular weight (Mn) was 5.970 × 10^4^ g/mol in the control, but 3.246 × 10^4^ g/mol in pCrcY, showing a 45.6% decrease. The weight-average molecular weight (Mw) also displayed a significant reduction, with 2.228 × 10^5^ g/mol in the control versus 9.468 × 10^4^ g/mol in pCrcY.

In contrast, under ONF and ONL conditions, no significant difference was observed between the pCrcY and control strains for all molecular weight parameters except Mp (Fig. 3), indicating that the impact of CrcY overexpression on PHA molecular weight may be carbon dependent.

All tested PHA samples exhibited melting temperatures (Tm) between 42 and 52 °C (Figure S10), consistent with the reported range for mcl-PHAs [38]. Only in the GNL-control PHA sample, additional high-temperature endotherms were detected at approximately 153 °C (β-phase) and 168 °C (α-phase), corresponding to distinct crystalline polymorphs of PHA. These α- and β-crystalline phases were absent in all other samples, including the PHA produced by pCrcY under the same GNL condition. It is possible that due to the different monomer composition of the two controls (Table 2), mcl-PHA produced from glucose shows higher crystallinity. Then, the overexpression of sRNAs in addition to reducing the Mw also appears to reduce the crystallinity. This effect appears to be absent in octanoate-grown cells, as the polymer already shows lower crystallinity (Figure S10).

### The effect of CrcY and CrcZ overexpression on the KT2440 proteome

**Fig. 4:**
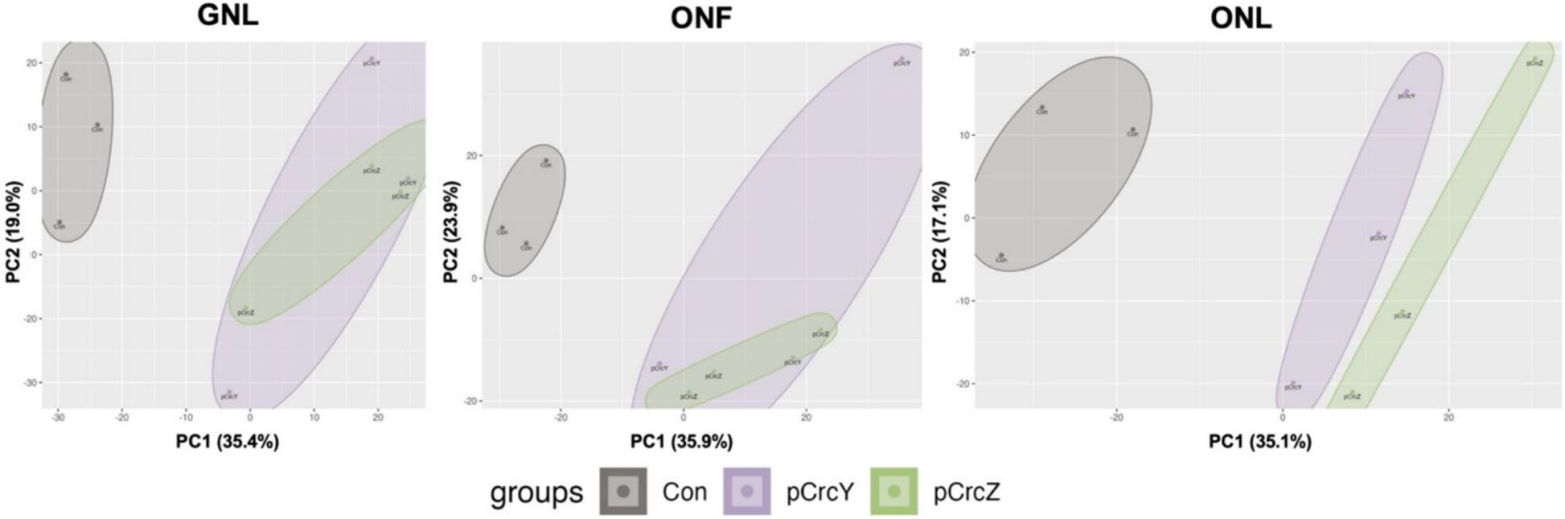
Principal component analysis (PCA) of pCrcY, pCrcZ, and the control (Con) strains' proteomes

It appears that the overexpression of either CrcY or CrcZ has a stimulating effect on PHA accumulation and can also alter the molecular weight of the produced PHA polymer. To explore the underlying mechanisms behind these effects, we performed a proteomic analysis of the overexpression strains under PHA-accumulating conditions, i.e. GNL, ONF, and ONL. Samples were taken at an early stationary phase: 21 h for GNL, 15 h for ONF, and 18 h for ONL conditions (Figure S1), when PHA metabolism was active and the synthesis of PHA exceeded its degradation [39]. During this period, the overexpression strains showed higher PHA levels compared to the control (Fig. S1). A total of 2036 proteins were detected across the three conditions. Principal component analysis (PCA) revealed significant differences in the proteomes between the control strain and the overexpression strains (Fig. 4). The overexpression strains show an overlap in the proteins responding to CrcY or CrcZ overexpression in GNL and ONF conditions, while a different response is observed in ONL conditions (Fig. 4).

KEGG pathway enrichment analysis of proteins with increased abundance revealed that ATP-binding cassette (ABC) transporters were significantly enriched under all tested conditions. ABC transporters are vital membrane proteins in bacteria, functioning beyond mere nutrient acquisition [40]. They contribute to preserving cell integrity, mediating adaptive responses to environmental stresses, and enabling intercellular communication. Not only are ABC transporters significant, but a variety of other transporters also showed significantly increased abundance in the pCrcY and pCrcZ proteomes, including those associated with the uptake of carbon, nitrogen, sulphate, iron, and phosphonate (Table S2).

The proteins involved in glucose uptake were also shown significant different abundance in pCrcY and pCrcZ proteomes. In *Pseudomonas*, glucose first enters the periplasmic space through the OprB-I (PP_1019) porin, which was downregulated 3.2- and 5.2-fold in pCrcY and pCrcZ strains under GNL conditions and was not detected under octanoate-supplied conditions (Table S3). Since OprB-I is a glucose-inducible protein [41], its lower abundance in the overexpression strains may reflect the depletion of glucose at the time of sampling. A faster glucose depletion compared to the control, along with higher total biomass, was observed in the overexpression strains (Fig. S2). This also explains its absence under octanoate conditions.

Glucose is then transported to the cytoplasm via the ABC transport system encoded by the *gstABCD* genes [42]. However, the expression levels of GstABCD in pCrcY and pCrcZ strains under the GNL condition showed no significant difference compared to the control. Interestingly, when octanoate was used, the ABC glucose transporter substrate-binding protein GtsA (PP_1015) was found to be over twofold more abundant in both pCrcY and pCrcZ strains under ONF and ONL conditions (Table S2). Additionally, GtsD (PP_1018), ATP binding subunit, showed a 1.2- and 1.7-fold increase in pCrcY and pCrcZ strains under ONF conditions but was not detected in the ONL proteome. Furthermore, PP_2264, annotated as a sugar transporter, was upregulated by 7.7- and 8.6-fold in pCrcY and pCrcZ strains under ONL conditions, 8.6- and 9.2-fold under ONF conditions, and 2.3- and 2.7-fold under GNL conditions compared to the control strain (Table S3).

It is worth mentioning that the *gstA*, *gstD*, *oprB-I* and *PP_2264* genes contain the CA motif needed for Crc/Hfq-dependent regulation. Interestingly, overexpression of CrcY or CrcZ led to increased expression of GstA, GstD, and PP_2264 proteins under octanoate-supplied conditions, while their expression showed inconsistent changes under GNL conditions in the pCrcY and pCrcZ strains.

**Fig. 5:**
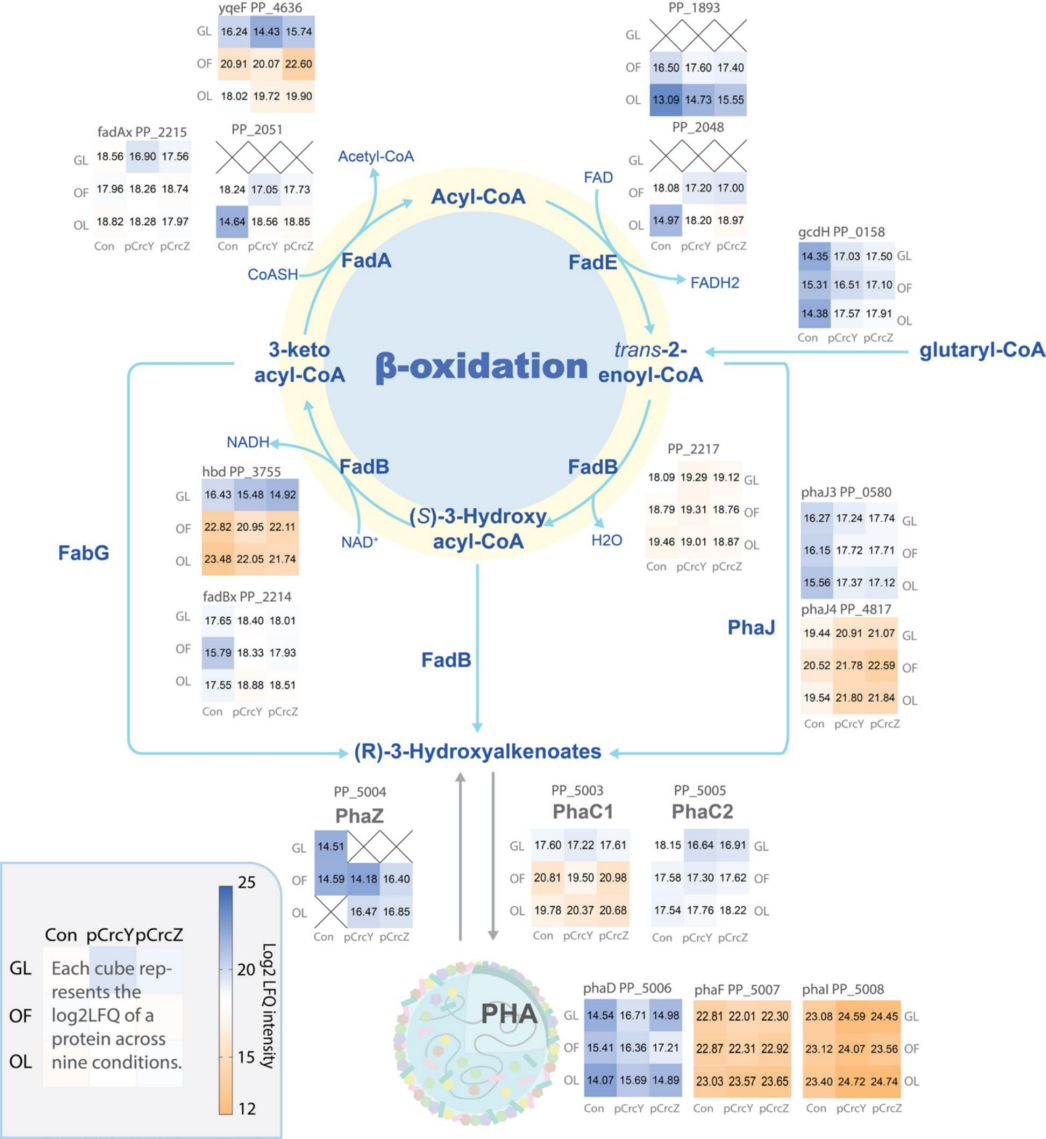
Differentially expressed proteins (DEPs) involved in the *β*-oxidation pathway and PHA operon. Each square represents the log₂LFQ intensity of a protein under three different culture conditions: glucose with nitrogen limitation (GL), octanoate with full nitrogen (OF), and octanoate with nitrogen limitation (OL). Data are shown for three strains: the control strain carrying the empty pBT’T plasmid (Con), and strains overexpressing either CrcY or CrcZ (pCrcY or pCrcZ). Blue tones indicate lower protein abundance, while orange tones indicate higher abundance. Values are mean (n = 3 biological replicates)

As mentioned, octanoate is catabolised via *β-*oxidation (Fig. 1). Neither fatty acid transporters nor acyl-CoA thioester synthetases showed significant differences in the pCrcY and pCrcZ strains compared to the control. However, among the annotated acyl-CoA dehydrogenases (ACADs) involved in the first oxidation step in KT2440 [43], two FadE homologs showed a significant differential abundance in the pCrcY and pCrcZ strains. Under ONL conditions, PP_2048 and PP_1893 were 3.2- and 4.0-fold higher in the pCrcY and pCrcZ strains, while PP_1893 was 2.9- and 2.5-fold higher in both strains. Under nitrogen-excess ONF conditions, the expression level of PP_1893 remained unchanged, while the expression of PP_2048 decreased by 1.5- and 1.1-fold in the pCrcY and pCrcZ strains (Fig. 5), respectively.

For PHA accumulation from related carbon sources, the next crucial step is the hydration of trans-2-enoyl-CoA intermediates into (*R*)-HA-CoAs, the direct substrates of PhaC. This reaction is catalysed by a *trans*-enoyl-CoA hydratase, of which three variants PhaJ1 (PP_4552), PhaJ3 (PP_0580), and PhaJ4 (PP_4817) have been identified in the genome of *P. putida* [44], with PhaJ4 as a key contributor to PHA monomer synthesis when C8 and C10 fatty acids are used as the carbon source [13]. PhaJ4 showed increased abundance in the overexpression strains in all conditions in this study (Fig. 5), and PhaJ3 exhibited elevated abundance in all conditions except GNL, while PhaJ1 did not show a significant difference compared to the control strain. Both PhaJ3 and PhaJ4 showed the most significant increase in abundance under ONL conditions, 1.81- and 2.30-fold, respectively, which corresponds to the highest increase in PHA level observed (Fig. 2B).

FadBA (PP_2136 and PP_2137), the main complex involved in the conversion of *trans*-2-enoyl-CoA into 3-keto-acyl-CoA [45], showed no significant difference in the overexpression strains. An additional FadBxAx complex (PP_2214 and PP_2215), which is believed to have less impact on PHA accumulation compared to the canonical FadA and FadB, showed condition-specific abundance changes in the overexpression strains. Under ONF conditions, FadBx (PP_2214) displayed more than a twofold increase in abundance in both pCrcY and pCrcZ strains (Fig. 5). In contrast, under GNL conditions, FadAx (PP_2215) exhibited 1.6- and 1.0-fold lower abundance in pCrcY and pCrcZ, respectively. No significant changes were observed for either protein under the other tested conditions.

The abundance level of PP_2051, which encodes acetoacetyl-CoA thiolase, exhibited 3.9- and 4.2-fold increases in the pCrcY and pCrcZ strains under ONL conditions, respectively. However, no significant changes were detected under ONF and GNL conditions. A similar pattern of expression changes was observed in FadE (PP_2048) since they both locate in the *β-*oxidation operon (PP_2047-PP_2051) [43]. The CrcY and CrcZ might not be the crucial regulators for this operon. Several other homologs of FadAB exhibited differential expression in the pCrcY and pCrcZ strains, including FadH (PP_2008), PP_2217, and Hbd (PP_3755). However, the expression patterns of FadB homologs in pCrcY and pCrcZ indicate no clear consistency across conditions, suggesting a complex regulatory mechanism influenced by varying environmental factors.

De novo fatty acid synthesis pathway, implicated in PHA synthesis from carbon-unrelated substrates, showed no change between the carbon sources (Table S3). When PHA non-related carbon sources are used, *Pseudomonas putida* is capable of synthesising PHA from acetyl-CoA via the fatty acid de novo pathway. However, when glucose, a non-PHA-related substrate, was used, the expression levels of fatty acid de novo pathway proteins did not show significant differences between the pCrcY and pCrcZ strains compared to the control strain that carried the empty plasmid.

Glutaryl-CoA dehydrogenase GcdH (PP_0158) plays a crucial role in the catabolism of lysine, hydroxylysine, and tryptophan through the oxidation of glutaryl-CoA to glutaconyl-CoA, which is subsequently decarboxylated to crotonyl-CoA, a direct substrate for PhaJ or FadB [46]. In pCrcY and pCrcZ strains, GcdH expression increased 2.5- and 3.0-fold under GNL conditions, 1.3- and 1.9-fold under ONF conditions, and 3.2- and 3.5-fold under ONL conditions. The gamma-glutamyl transpeptidase GGT (PP_4659), a key enzyme in the glutathione cycle that hydrolyses glutathione (GSH) into L-glutamate and L-cysteinylglycine [47], was found to contain a CA motif near its start codon. This enzyme showed over threefold increased abundance in both the pCrcY and pCrcZ under ONF and ONL conditions. GGT catalysis is essential for recycling glutathione components.

While we expected to see a difference in the abundance of PHA polymerases PhaC1 (PP_5003) and PhaC2 (PP_5005), especially PhaC1 which was identified as a target gene regulated by Crc [31], no significant difference was observed when either pCrcY or pCrcZ was overexpressed compared to the control (Fig. 5).

It was previously shown that PhaZ depolymerase is needed for optimal PHA metabolism [14, 48], optimising the carbon and energy flow. Inconsistent changes were observed for overexpression strains under different growth conditions (Fig. 5). Under ONL condition, PhaZ was exclusively detected in the overexpression strains, and the expression was 1.8-fold higher in the overexpression strains compared to the control in ONF condition. However, in GNL condition, PhaZ was exclusively detected in the control strain. This inconsistency in PhaZ expression could be the result of sampling points, and it makes it difficult to directly link it to the overexpression of the two sRNAs. Additionally, other proteins in the PHA cluster, including PhaI (PP_5008) and PhaD (PP_5006), also exhibit inconsistent changes in their expression levels.

### The double deletion of *crcY* and *crcZ* significantly decreases PHA accumulation

**Fig. 6:**
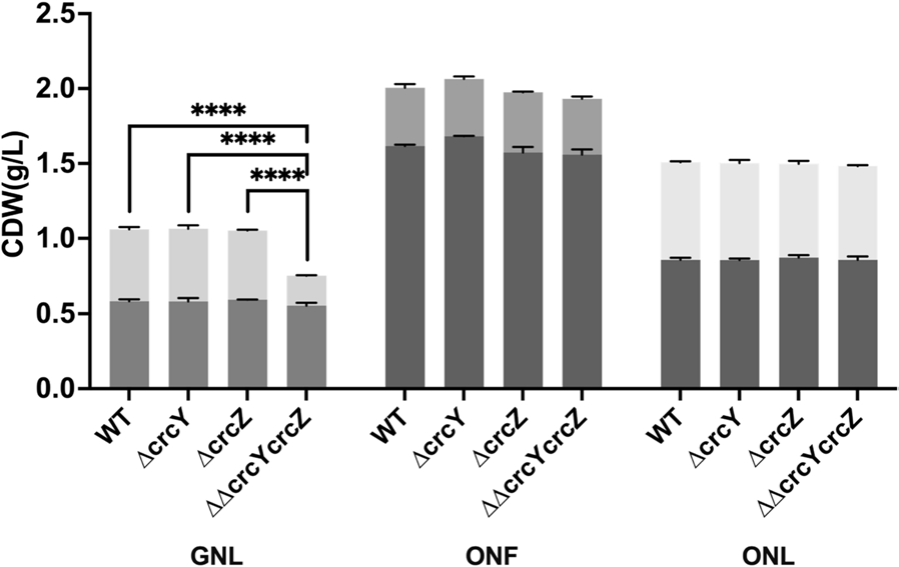
Biomass and PHA accumulation in *P. putida* KT2440 CrcY and CrcZ deletion strains. The KT Δ*crcY* and KT Δ*crcZ*, the double deletion strain KT ΔΔ*crcYcrcZ*, and the wild-type KT2440. The strains were cultivated for 48 h in MSM supplemented with glucose (G) and octanoate (O), with nitrogen conditions indicated as nitrogen-full (NF) and nitrogen-limited (NL). PHA amounts are represented by upper bars, while biomass (excluding PHA) is shown by bottom bars. Asterisks indicate significant differences between the samples and the control, or the comparison indicated in brackets: * P ≤ 0.05, ** P ≤ 0.01, *** P ≤ 0.001, **** P ≤ 0.0001

While the in *trans* overexpression of CrcY or CrcZ has a positive effect on PHA metabolism, it is not clear if these two sRNAs are needed for PHA accumulation. Secondly, it is not clear if the two sRNAs could have a complementary role in PHA accumulation. Single deletion strains for CrcZ or CrcY were created and their PHA-accumulating capacity was analysed. Neither KT Δ*crcY* nor KT Δ*crcZ* showed a difference compared to the wild-type (WT) KT2440 in terms of growth or PHA level achieved after 48 h cultivation in the sole carbon source media (Fig. 6A). Under all tested conditions, Δ*crcY* and Δ*crcZ* exhibited slight changes in PHA-free biomass, but the overall changes were within ± 10%. The individual loss of CrcY or CrcZ does not substantially alter PHA accumulation, and it may be that the absence of one sRNA leads to a compensatory increase in the levels of the remaining one [26]. Therefore, CrcY and CrcZ could play complementary roles in PHA accumulation. When both CrcY and CrcZ were deleted, the KT ΔΔ*crcYcrcZ* strain exhibited a 60% decrease in PHA yield under the GNL condition compared to the WT (Fig. 6A). No differences were observed under the ONF and ONL conditions, which is interesting, as the overexpression of either of the sRNAs leads to the highest increase in PHA under these two conditions.

Overall, it appears that CrcY or CrcZ is not essential to PHA accumulation, and only when both are removed can a significant decrease in PHA level be observed when glucose is supplied. The complementation strains demonstrate that the presence of CrcY and CrcZ supports PHA accumulation under glucose-consuming conditions. Values are mean ± SD (n = 3 biological replicates).

### The effect of CrcY and CrcZ on PHA metabolism depends on the Hfq and Crc proteins

**Fig. 7:**
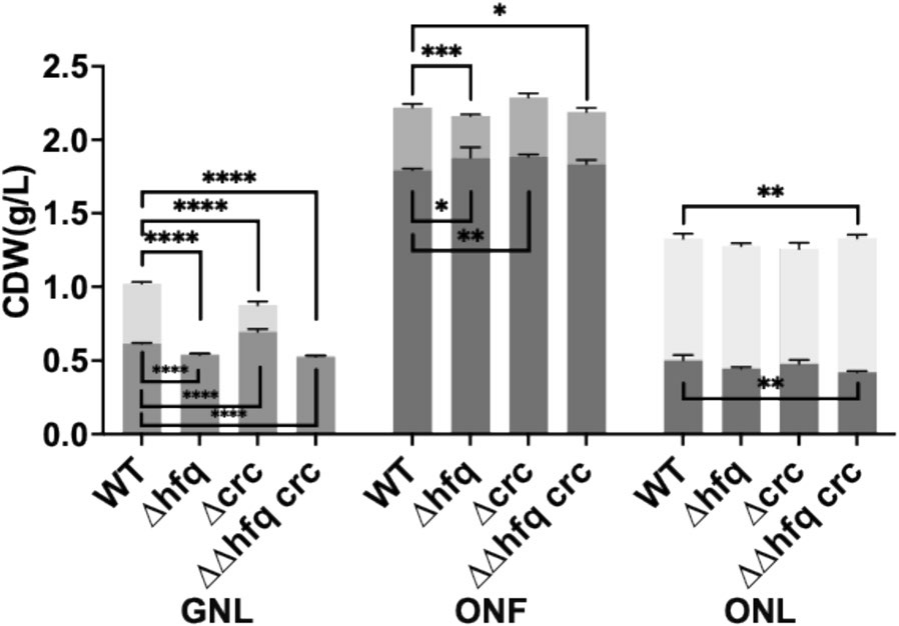
Biomass and PHA accumulation in *P. putida* KT2440 Hfq and Crc deletion strains. The KT Δ*hfq* and KT Δ*crc*, the double deletion strain KT ΔΔ*hfq crc*, and the wild-type KT2440, strains were cultivated for 48 h in MSM supplemented with glucose (G) and octanoate (O), with nitrogen conditions indicated as nitrogen-full (NF) and nitrogen-limited (NL). PHA amounts are represented by upper bars, while biomass (excluding PHA) is shown by bottom bars. Asterisks indicate significant differences between the samples and the control, or the comparison indicated in brackets: * P ≤ 0.05, ** P ≤ 0.01, *** P ≤ 0.001, **** P ≤ 0.0001. Values are mean ± SD (n = 3 biological replicates)

As described above, CrcY and CrcZ are only one component of the CCR system. To test whether their effects on PHA metabolism require other CCR elements, we generated deletion strains for *crc* and *hfq*, and overexpressed the sRNAs *in trans* in these backgrounds. Removing Hfq had the most profound effect when unrelated carbon substrate, i.e. glucose was used, and this was combined with nitrogen limitation (Fig. 7). No PHA was detected in KT Δ*hfq* in GNL conditions (Fig. 7). The total biomass was 1.9-fold lower than the wild-type, and this reduction was due to both a lack of PHA mass and a 12.3% decrease in PHA-free biomass compared to the wild-type (Fig. 7). KT Δ*hfq* remained capable of accumulating PHA when octanoate was used as a carbon and energy source. However, PHA yield was reduced by 33.1% in ONF conditions, while PHA level was unaffected in ONL condition (Fig. 7).

The deletion of *crc* (KT Δ*crc*) led to a 2.3-fold reduction in PHA in GNL, while no effect was observed when octanoate was used regardless of the amount of nitrogen supplied. When both *hfq* and *crc* genes were deleted from wild-type KT2440, the KT ΔΔ*hfq crc* strain exhibited the same phenotype and PHA accumulation levels as the Δ*hfq* mutant (Fig. 7). Under ONL conditions, however, KT ΔΔ*hfq crc* exhibited minor changes, with a 16.2% decrease in PHA-free biomass and a 10.0% increase in PHA accumulation.

**Fig. 8:**
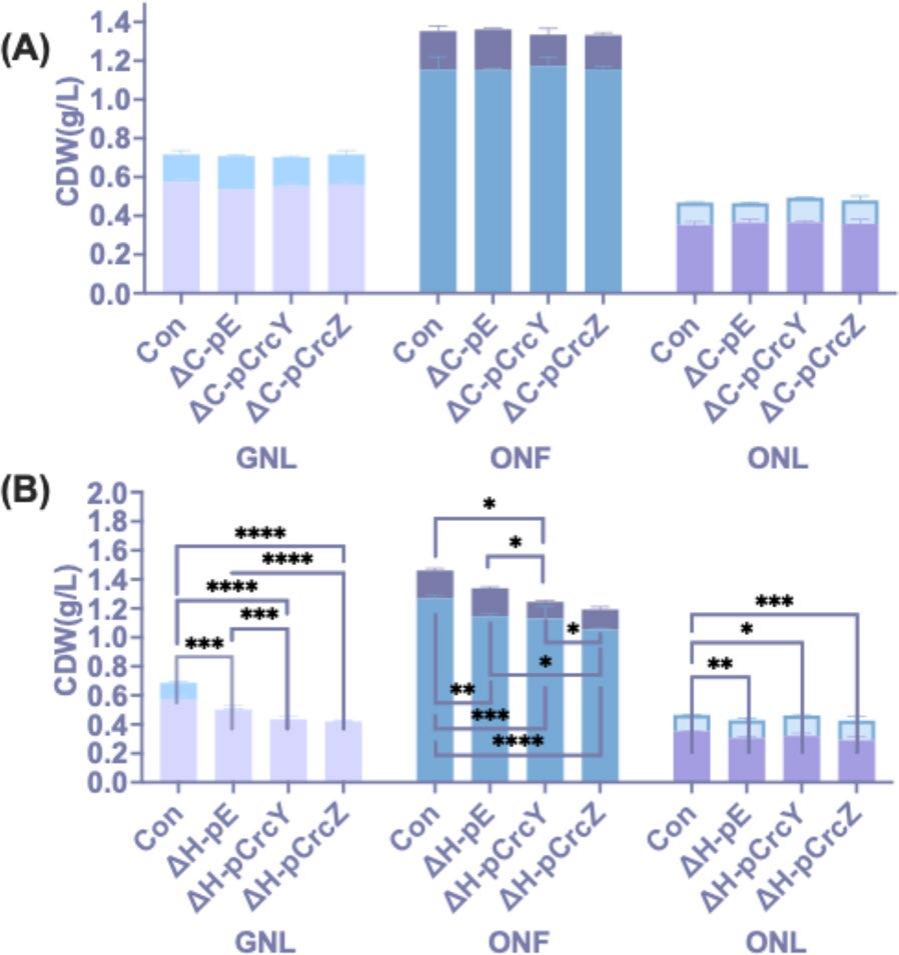
Effect of CrcY and CrcZ overexpression on biomass and PHA accumulation in Δ*crc* and Δ*hfq*. **A** Influence of small RNA CrcY and CrcZ overexpression on biomass and PHA accumulation in KT Δ*crc*. **B** Influence of small RNA CrcY and CrcZ overexpression on biomass and PHA accumulation in KT Δ*hfq*. pBT’Tmcs vector was used for the *in trans* overexpression of CrcY (pBT'T-crcY; pCrcY), or CrcZ (pBT'T-crcZ; pCrcZ). The KT2440 strains transformed with an empty pBT'T was used as a control (con). The strains were cultivated for 48 h in MSM supplemented with glucose (G) and octanoate (O), with nitrogen conditions indicated as nitrogen-full (NF) and nitrogen-limited (NL). PHA amounts are represented by lighter-coloured bars, while biomass (excluding PHA) is shown by darker-coloured bars. Asterisks indicate significant differences between the samples and the control, or the comparison indicated in brackets: * P ≤ 0.05, ** P ≤ 0.01, *** P ≤ 0.001, **** P ≤ 0.0001. Values are mean ± SD (n = 3 biological replicates)

Next, CrcY and CrcZ were overexpressed in KT Δ*hfq* or KT Δ*crc*. The overexpression of CrcY and CrcZ in KT Δ*crc* (to yield ΔC-pCrcY and ΔC-pCrcZ) resulted in no significant differences in total biomass or PHA amount compared to the controls (Fig. 8A), suggesting the effect of sRNAs on PHA accumulation requires Crc. The presence of Hfq is equally important, as the Δ*hfq* overexpression strains (ΔH-pCrcY and ΔH-pCrcZ) showed no increase in PHA yield compared to the controls with glucose as a substrate (Fig. 8B). ΔH-pCrcY, ΔH-pCrcZ, or the KT Δ*hfq* with an empty pBT’Tmcs vector (ΔH-con) showed no PHA accumulation in GNL condition (Fig. 8B). When octanoate was used (ONL), ΔH-pCrcY, ΔH-pCrcZ, and ΔH-con showed no difference in PHA and minor changes (± 5%) in non-PHA biomass compared to the wild-type empty vector control in ONL condition (Fig. 8B).

Under ONF conditions, the ΔH-pCrcY, ΔH-pCrcZ, and ΔH-pE strains showed approximately a 10% decrease in non-PHA biomass compared to the control, suggesting this reduction resulted from the loss of Hfq. The ΔH-pE strain and the control accumulated similar amounts of PHA after 48 h of incubation, reaching 0.19 g/L in ONF conditions. However, ΔH-pCrcY and ΔH-pCrcZ exhibited 41.19% and 27.69% reductions in PHA yield, respectively, compared to the control (Fig. 8B). The absence of Hfq under ONF conditions, combined with the relatively high levels of free CrcY and CrcZ, negatively affected PHA accumulation. The enhancement of PHA production through the overexpression of CrcY or CrcZ relies on a fully functional CCR system in *Pseudomonas*. Achieving positive effects on PHA yields is challenging by simply removing the repressors from the internal environment. On the contrary, an imbalance in regulatory factors tends to negatively impact both growth and PHA metabolism.

### Discussion

The advantages PHAs have over other polymers are widely known, such as biodegradability, biocompatibility, and avoidance of fossil feedstocks in production. However, PHAs are equally known for mechanical properties that do not compare well to petrochemical counterparts. Approaches to overcome these limitations include using plasticisers, polymer blending, or chemical modification of PHAs [49]. These modifications have to be done in a way not to compromise the biodegradability and biocompatibility of PHAs. If two PHA polymers, polyhydroxybutyrate (PHB) and an mcl-PHA polyhydroxyoctanoate (PHO), are blended, the resulting material appears to have higher ductility and improved thermal stability [50]. Simultaneously, this does not compromise the biodegradability of the polymer [51].

In this study, we have linked the elements of carbon catabolite repression (CCR) to mcl-PHA metabolism in KT2440 and showed that by overexpressing small RNAs CrcY and CrcZ the mcl-PHA yield can be boosted between 1.3- and 3.5-fold (Fig. 2). It is likely that CrcY and CrcZ have a complementary role, as suggested by the results where the overexpression of either CrcY or CrcZ had a similar positive effect on PHA accumulation. In addition to increasing the PHA level, the overexpression of either CrcY or CrcZ leads to a decrease in the Mw of the produced polymer. The Mw of an additive plays a significant role in modulating a polymer’s properties [52].

A previous study in *P. putida* KT2442 reported that deletion of the *crc* gene increased PHA production in LB medium supplemented with octanoic acid but had no effect in minimal medium and further demonstrated that Crc exerts post-transcriptional inhibition on the PHA synthase PhaC1 [31]. Increased levels of CrcY or CrcZ have been shown to relieve Hfq/Crc-dependent catabolite repression by sequestering the Hfq/Crc complex, thereby reducing its ability to bind target mRNAs [26, 53]. Based on this mechanism, we initially hypothesised that overexpression of CrcY and CrcZ would relieve Hfq/Crc-mediated inhibition (e.g., enhance *phaC1* expression) and thereby promote PHA synthesis. It was further shown that when either of the sRNAs was overexpressed in a *crc*- or *hfq*-deletion background, no increase in PHA accumulation was observed (Fig. 8), indicating that the presence of an intact CCR system is required for the observed phenotype, i.e. increased PHA accumulation.

Having in mind the standard explanation of the CCR mechanism [21, 24–26], it would be expected that the removal of the Hfq/Crc regulators should mimic the effect of CrcY and CrcZ overexpression, as both are expected to relieve CCR inhibition. In contrast, deletion of *crc, hfq*, or the combined *crc hfq* strain resulted in markedly reduced PHA accumulation (17.0% to 98.4% decrease), with the most severe effect observed in the *Δhfq* mutant under GNL conditions, where PHA accumulation was completely abolished and residual biomass was significantly reduced compared with the wild type (Fig. 7).

Furthermore, in agreement with the above-mentioned classical explanation of CCR, and aligned to our results that the overexpression of *crcY* and *crcZ* would relieve Hfq/Crc-dependent repression and thereby enhance PHA production, the deletion of these sRNAs would strengthen repression and reduce PHA yields. However, our results showed that the deletion of *crcY* and *crcZ* did not consistently repress PHA accumulation across all tested conditions. A pronounced effect was only observed under GNL, where simultaneous deletion of both sRNAs reduced PHA yield by 60%. The complementary roles of CrcY and CrcZ were further demonstrated by the absence of a phenotype in single-deletion strains under GNL (Fig. 6). These findings indicate that CrcY and CrcZ are not essential for PHA accumulation, and that the presence of either one is sufficient to maintain wild-type levels. Moreover, while lighter CCR inhibition generally supports enhanced PHA accumulation across tested conditions, stronger CCR is not detrimental for PHA accumulation except under GNL conditions.

All *crcY/crcZ* and *crc hfq* deletions had only minor effects on PHA accumulation under octanoate-supplied conditions (ONF and ONL), but significantly reduced PHA production under GNL. These findings suggest that the impact of CCR element deletions on PHA accumulation is carbon-substrate dependent and highlight their central role in glucose metabolism. To determine whether this effect is restricted to PHA-accumulating conditions, the deletion strains were also cultivated with glucose under nitrogen-replete conditions (GNF). In this case, *Δhfq* and *ΔΔhfq crc* showed nearly 30% lower biomass than the wild type (Fig. S3), confirming the critical role of Hfq in glucose metabolism, whereas the effects of *crcY/crcZ* or *crc* deletions were negligible under these conditions.

The substrate-dependent effects may be explained by the distinct metabolic pathways used for glucose and octanoate (Fig. 1). To date, no direct Hfq/Crc targets have been reported in fatty acid metabolism, whereas several targets related to glucose uptake and catabolism have been identified. In *P. putida* KT2440, Hfq/Crc binding sites were found in the translation initiation regions of *gltR-2* (encoding the activator of the *gstABCD–oprB-I* operon), *gstA* and *gstD* (encoding glucose ABC transporters), and *oprB-I* (encoding the glucose porin). Similarly, in *P. stutzeri* A1501, genes related to glucose uptake and metabolism—including *gltR* (transcriptional regulator), *oprB* (glucose porin), and *zwf* (glucose-6-phosphate dehydrogenase)—were identified as Hfq/Crc regulon members [54]. Notably, in *P. putida* KT2440, deletion of *crcY* and *crcZ* reduced the mRNA levels of glucose uptake genes such as *oprB-I* and *gstABCD* [55]. This supports a role for the CCR system in coordinating glycolytic and gluconeogenic metabolism in *Pseudomonas*. Consistent with this view, deleting CCR elements is likely to disrupt translation of Hfq/Crc targets involved in glucose catabolism: in our study, *hfq* deletion impaired growth on glucose, a phenotype also reported in *P. stutzeri* [57] and in *Bacillus subtilis* [58]. The specific contribution of Hfq to glucose utilisation—and its connection to PHA metabolism—remains to be elucidated.

To investigate why in trans overexpression of CrcY and CrcZ boosts PHA accumulation, we carried out label-free quantitative proteomics of pCrcY/pCrcZ and control strains under PHA-accumulating conditions. Based on our working hypothesis, *in trans* overexpression of CrcY and CrcZ should attenuate Hfq/Crc-mediated repression and thus increase the abundance of proteins encoded by putative Hfq/Crc targets carrying the CA motif (AAnAAnAA) near the ribosome-binding site. Contrary to expectation, many CA-motif-bearing candidates did not show higher abundance in the pCrcY or pCrcZ proteomes relative to the control (Fig. S7). Notably, although the motif is present in *phaC1* and in the phasin genes *phaI* and *phaF*, none of these proteins increased in abundance. This suggests that the higher PHA titres observed in the sRNA-overexpression strains are unlikely to be driven by enhanced translation of *phaC1*, while other sRNA-dependent interactions with these genes cannot be excluded. Similarly, although *oprB-1*, *gstA*, and *gstD* harbour CA motifs and are plausible CCR targets, GstA and GstD showed no change in abundance in pCrcY and pCrcZ under GNL, whereas both were elevated under octanoate conditions. Taken together, the data are inconsistent with a simple schema in which the presence of a CA motif entails Hfq/Crc-mediated repression that is universally alleviated by CrcY/CrcZ overexpression. Target responses are strongly condition-dependent and are likely shaped by additional regulatory layers.

However, the direct targets of the two sRNAs investigated here remain unclear. The main pathways surveyed in the overexpression strains were the ones associated with the carbon and energy substrate supplied, i.e. Entner–Doudoroff (ED) pathway for glucose oxidation in KT2440 and *β-*oxidation for fatty acid catabolism, Krebs cycle, and PHA synthesis pathways. To be noted, many proteins that show increased abundance when two sRNAs are overexpressed on a DNA level lack CA motifs, suggesting that they may not be under direct CCR regulation (Fig. S9; Table S3).

The *in trans* overexpression of CrcY and CrcZ led to a broad increase in the abundance of transporters, especially ABC transporters. A similar expression trend was observed in other analyses, where ABC transporters were found to be significantly more abundant under PHA-producing conditions compared to non-PHA-producing conditions [57], regardless of whether nitrogen or phosphorus was used to boost PHA accumulation [58]. Although the link between ABC transporter expression and PHA accumulation remains unclear, it may be part of the stress response to nutrient limitation used for PHA production or contribute to the maintenance of cell integrity. In our study, the pCrcY and pCrcZ proteomes exhibited a transporter expression pattern consistent with PHA-producing proteomes, with however higher abundance of transporters in pCrcY and pCrcZ compared to Con (KT2440 pBT’T grown under PHA-accumulating conditions), suggesting a potential link between these sRNAs and higher transporter presence.

KT2440 uses predominantly the ED pathway and, to a low extent, the pentose phosphate (PP) pathway to oxidise glucose due to the absence of 6-phosphofructo-1-kinase (Pfk) [59]. Other than the decrease in some of the glucose transporters (Fig. S9), which is likely a consequence of the depleted glucose in the medium observed in these experiments, no changes in either of the alternative routes for glucose catabolism were observed in the overexpression strains grown with glucose and in PHA-accumulating conditions (GNL). There were no consistent changes in the abundance of proteins related to the TCA cycle or de novo fatty acid synthesis employed for the PHA monomer synthesis when unrelated substrates, i.e. glucose are used (Fig. S9).

One of the enzymes that showed increased abundance upon the overexpression of CrcY or CrcZ in GNL conditions, but also in both ONL and ONF, is GcdH. The abundance increase was more pronounced under nitrogen limitation (Fig. 5). This enzyme oxidatively decarboxylates glutaryl-CoA, a product of the catabolism of tryptophan, lysine, and hydroxylysine, into crotonyl-CoA, which ultimately is converted into two acetyl-CoA molecules. This could contribute to the increased pool of this key intermediate, used as a starting molecule in de novo fatty acid synthesis and PHA synthesis (Fig. 1).

The increase of PHA with octanoate as a carbon and energy substrate could come from the increased abundance of one of the FadE homologues, PP_1893, oxidising octanoyl-CoA to *trans*-2-enoyl-CoA, and PhaJ homologues (PP_0580 and PP4817), involved in the hydration of *trans*-2-enoyl-CoA to (*R*)-3-hydroxyoctanoyl-CoA, a PHA monomer (Fig. 5). The overexpression of PhaJ was shown to increase PHA content in *P. putida* KCTC1639, from 18 to 27% [60].

While *β*-oxidation is the main pathway supplying PHA monomers when fatty acids are used as a carbon and energy substrate, the de novo fatty acid synthesis pathway also contributes to the synthesis of PHA monomers, as evidenced by the presence of C10 and C12 monomers when octanoate, a C8 fatty acid, is fed (Table 2, [13]). No change in PHA monomer composition was observed in pCrcY and pCrcZ compared to Con, suggesting that there was no change in the distribution of fluxes through de novo fatty acid synthesis and *β*-oxidation. In addition to GcdH abundance increase when octanoate was used, we have observed over a threefold increase in abundance in gamma-glutamyl transpeptidase GGT, which hydrolyses glutathione to L-glutamate and L-cysteinylglycine. Glutathione is one of the key molecules that is involved in the redox balance homeostasis through the glutathione cycle. Recently, a direct link between an increased pool of NADPH as a response to oxidative stress and the increased glutathione pool was demonstrated [61]. As PHA represents a major NADPH sink, it is possible that when the sRNAs are overexpressed and the PHA level increases, the increased abundance of GGT would mean an increased cleavage of glutathione, thereby optimising the cell’s resource utilisation.

Although overexpression of *crcY* and *crcZ* enhanced PHA production in an Hfq- and Crc-dependent manner, the associated proteome-wide responses extended well beyond the currently recognised Hfq/Crc regulon. It could reveal that apart from a role as CCR antagonists, there are additional roles for CrcY and CrcZ in the regulatory network. CrcZ was identified as a target within the Rsm system, demonstrating that RsmN and RsmA bind differentially to specific regions of CrcZ [62]. In our study, although pCrcY and pCrcZ shared similar proteomic profiles, distinct sets of DEPs were uniquely upregulated or downregulated in each strain (Figure S5 and S6). This reflects differences in the specificity of sRNA CrcY and CrcZ’s regulatory effects, which warrant further investigation. Two transcriptional regulators (PP_4429, PP_0019) were exclusively detected in the pCrcY strain under nitrogen-limited conditions (GNL and ONL), indicating potential novel regulatory functions of CrcY. There are still unexplored mechanisms and roles for these two small RNAs, highlighting the need for further investigation.

Notably, protein abundances in pCrcY and pCrcZ differed across carbon and nitrogen regimes. Two factors likely contribute to this: (i) glucose and octanoate enter distinct native pathways (e.g., glycolysis vs *β-*oxidation), creating different metabolic backgrounds that modulate target responsiveness; and (ii) basal expression of *crcY*/*crcZ* varies between glucose and octanoate and between nitrogen-replete and nitrogen-limited states, establishing different CCR intensities under each condition. At present, however, CCR “strength” remains difficult to quantify rigorously. Because Crc lacks mRNA-binding activity [32, 33], Hfq is the domain regulator in the CCR system; however, the complexity of Hfq-mediated regulation has been underestimated in CCR-related studies in *Pseudomonas* strains. In bacteria, Hfq is a conserved RNA-binding chaperone of the Sm/LSm family with a ring-like oligomeric architecture that presents multiple, non-equivalent RNA-binding surfaces [63–65]. It facilitates base-pairing between sRNAs and target mRNAs and thereby modulates translation initiation (repression or activation), RNA stability (protection or RNase E–mediated cleavage), and RNA turnover via polyadenylation-coupled decay [66]. Hfq functions as a core hub of global post-transcriptional networks, with model organisms such as *Escherichia coli* and *Salmonella enterica* expressing ~ 100 sRNAs [67, 68]. In vivo, Hfq dynamically engages diverse RNAs, and because sRNAs and mRNAs compete for a limited Hfq pool, protein availability becomes an important determinant of regulatory outcomes [69]. However, in *P. putida*, the Hfq–RNA interactome remains poorly characterised. Direct quantification of Hfq and of *crcY*/*crcZ* expression is unlikely to reflect functional Hfq availability, nor to provide a faithful proxy for overall CCR ‘strength’. Moreover, existing CCR models rarely consider the complexity of intracellular regulatory networks or the condition-specific metabolic background that reshapes transcriptional and translational profiles. This may help explain why sRNA overexpression does not consistently derepress putative targets under all conditions. Nevertheless, the precise mechanisms of CCR, as well as its interplay with other regulatory networks, remain to be fully elucidated.

Overall, the initial hypothesis proved incomplete; this study highlights the complex interplay between CCR, small RNAs, and PHA metabolism in *P. putida* KT2440. The findings provide valuable insights into metabolic regulation that can be leveraged for biotechnological applications, particularly in optimising PHA production through genetic engineering.

## Materials and methods

### Strains, media, and culture conditions

The strains used in these studies are described in Table S1. *E. coli* DH5α and *E. coli* λ pir cultures were grown in LB Broth (lysogeny broth) medium (Sigma-Aldrich) at 37°C, 200 rpm, while *P. putida* was grown at 30°C.

To test the polyhydroxyalkanoate (PHA) accumulation in *P. putida* strains, they were cultivated in Minimal Salts Medium (MSM) [70]: per litre 9 g Na_3_PO_4_∙12H_2_O, 1.5 g KH_2_PO_4_, and 1 g NH_4_Cl (non-limited conditions; nitrogen-full MSM_full_) or 0.25 g NH_4_Cl (nitrogen-limiting condition; MSM_lim_), 1 mM MgSO_4_∙7H_2_O, and 1 mL trace elements (per litre: 4 g ZnSO_4_∙7H_2_O; 1 g MnCl∙4H_2_O; 0.2 g Na_2_B_4_O_7_∙10H_2_O; 0.3 g NiCl_2_∙6H_2_O; 1 g Na_2_MoO_4_∙2H_2_O; 1 g CuCl∙2H_2_O; 7.6 g FeSO_4_∙7H_2_O; added after autoclaving). After autoclaving, the medium was supplemented with a carbon and energy source to a final carbon concentration of 1.95 g of carbon per litre (g_c_/L) [71]: sodium octanoate (referred to as octanoate in the main text, 3.376 g/L) or glucose (4.875 g/L).

When needed, the medium was supplemented with kanamycin (50 µg/mL, VWR International), gentamicin (35 µg/mL, Thermo Scientific), and tetracycline (10 µg/mL for *E. coli*; 25 µg/mL for *P. putida*, Thermo Scientific).

Unless otherwise stated, all experiments were performed with three independent biological replicates. Data are presented as mean ± standard deviation (SD).

### Generation of *P. putida* KT2440 mutants

The genes of interest, including crcY (PP_mr44), crcZ (PP_mr53), crc (PP_5292), hfq (PP_4894), in *P. putida* KT2440 were scarlessly deleted using modified CRISPR/Cas9 systems and methodology [72]. The work-flow of gene deletion is published in a previous protocol [73]. For *in trans* expression of the target genes, pBT’Tmcs plasmid was used [74]. The plasmids were transformed into *P. putida* KT2440 wild-type and deletion mutants via electroporation, and the transformants were selected with kanamycin. Plasmids used in this work are listed in Table S1**.** Primers used in this study can be found in Table S1**.**

### PHA incubation, extraction, and content determination

A single colony of *P. putida* KT2440 strain from LB plate was inoculated in 3 mL of LB medium and cultivated for ~ 16 h at 30 °C, 200 rpm. 1 mL of the overnight culture was inoculated into 50 mL of MSM_full_ in a 250-mL conical flask with 1.95 g_c_/L of sodium octanoate (3.376g/L) or glucose (4.875 g/L), and incubated at 30°C, with shaking at 200 rpm for 16 h.

The seed culture was diluted with MSM_full_ medium to get an OD_600nm_ of 1, after which 1 mL of diluted culture was inoculated into 50 mL MSM_full_ or MSM_lim_ supplemented with 1.95 g_c_/L of carbon and energy source in 250-mL conical flasks. After 48 h of incubation, cells were harvested by centrifugation at 13000 rpm at 4 °C for 10 min. The cell pellet was washed with 1 mL of phosphate buffer (9 g/L Na_3_PO_4_∙12H_2_O, 1.5 g/L KH_2_PO_4_, pH = 7.0) and freeze-dried.

The polymer content was assayed by subjecting the lyophilised cells to acidic methanolysis as previously described [75]**.** The PHA monomers’ methylesters were assayed by GC using a Hewlett-Packard 6890N chromatograph equipped with a HP-Innowax capillary column (30 m × 0.25 mm, 0.50 μm film thickness; Agilent Technologies) and a flame ionisation detector (FID), using the temperature program previously described [75]**.** Total PHA content was determined as a percentage of CDW.

### Proteomic studies

Proteomic sample preparation and LC–MS/MS analysis were performed as previously described [76], with minor modifications. Briefly, cultures were harvested at early stationary phase under GNL, ONL, and ONF conditions, washed with chilled phosphate buffer, and stored at −80 °C. Cell pellets were lysed in 8 M urea, reduced, alkylated, and digested overnight with trypsin. Peptides were purified using C18 ZipTips and analysed on a timsTOF Pro mass spectrometer (Bruker Daltonics) coupled with the EvoSep One system using the 30 samples/day method. Raw MS data were processed using MaxQuant [77], and statistical analysis of label-free quantification (LFQ) intensities was performed on Perseus [78] and the amica web platform [79]. Differentially expressed proteins (DEPs) were identified based on log₂ fold change ≥ 1 and p < 0.05. KEGG enrichment and visualisation analyses were conducted using clusterProfiler [80] and UpSetR [81]. Full experimental details and protocol are available in the supplement file.

### PHA characterisation

Laboratory-scale batch bioreactor experiments were conducted in a 5 L Biostat B stirred-tank bioreactor vessel (Sartorius, Germany) with a 3 L working volume to produce PHA.

A single colony of the *Pseudomonas putida* KT2440 strain carrying the pBT’T-crcY plasmid was inoculated into 6 mL of LB medium and cultivated for approximately 16 h at 30 °C with agitation at 200 rpm. This overnight culture (6 mL) was subsequently transferred into 300 mL of MSM medium in a 500 mL conical flask containing either 1.95 g_c_/L (equivalent to 3.376 g/L) of sodium octanoate or 4.875 g/L of glucose. The culture was incubated at 30 °C with shaking at 200 rpm for 16 h. All media used were supplemented with 50 µg/mL kanamycin.

The culture medium, designed to maintain the same C:N ratio as the MSM medium used in flask experiments but with a higher total carbon content, contained 9 g/L Na₃PO₄∙12H₂O, 1.5 g/L KH₂PO₄, 8.31 g/L sodium octanoate, and either 2.46 g/L NH₄Cl (octanoate nitrogen-full condition) or 0.616 g/L NH₄Cl (octanoate nitrogen-limited condition). For glucose nitrogen-limited conditions, 30 g/L glucose was used with 1.54 g/L NH₄Cl. All ingredients, except for the carbon source, were dissolved in deionised (DI) water directly in the bioreactor vessel and autoclaved, along with the dissolved oxygen (DO) and pH probes. Carbon sources were sterilised separately as 10 × concentrated solutions. After autoclaving, 3 mL of 1 M MgSO₄∙7H₂O, 3 mL of trace elements, 3 mL of 50 mg/mL kanamycin, and the sterilised carbon source solutions were added to the medium. The 300 mL flask culture was used to inoculate the bioreactor containing 3 L of culture medium.

Cultures were carried out with an air supply rate of 3 L/min and an initial agitation rate of 500 rpm. These parameters were adjusted as necessary during culture growth to maintain aerobic conditions, with increased agitation and air supply as required. The medium pH was maintained at 7.0 through the automatic addition of 1 M sodium hydroxide (NaOH) or 15% sulfuric acid (H₂SO₄). The temperature was controlled at 30 °C. Foam formation was managed manually by adding a 1:10 dilution of Antifoam-Y emulsion (Sigma-Aldrich, Ireland) as needed. Cells were harvested by centrifugation at 13,500 rpm for 10 min at 4 °C, followed by freeze-drying for further analysis.

PHA extraction was performed using a Soxhlet extractor. Up to 10 g of freeze-dried cell biomass was placed in a filter within the extraction chamber. Chloroform (200 mL) was added to the round-bottom flask, which was heated to 60 °C using a heating mantle. The extraction was carried out for 2 h, during which chloroform vaporised, condensed, and percolated through the biomass, dissolving the PHA. After the extraction, the residual cell biomass was removed and discarded. The chloroform was evaporated in a rotavapour under vacuum (Buchi, Switzerland). The polymer was solvent cast in a petri dish and was dried to constant weight in a fume hood; the dried polymer was weighed and used for further analysis.

Molecular weights of PHA samples were determined using an Agilent 1260 Infinity II Multi-Detector Gel Permeation Chromatography (GPC) system equipped with RUI and diode-array detectors, and the columns were packed with PLgel 10 µm MIXED-B beads. The GPC used HPLC grade chloroform at 35 °C at a flow rate of 1 ml min^−1^. Stock PHA (20 mg) was dissolved in HPLC grade chloroform (10 ml) at room temperature for 45 min; 2 ml of stock solution was taken for GPC analysis. The solution was filtered through a 0.24 µm PTFE filter.

Differential Scanning Calorimetry (DSC) experiments were run on a TA Instruments DSC 2500 with nitrogen and N5-Oxygen gas capacities. All tested samples weighed between 2–12 mg and were tested in Tzero aluminium pans with Tzero hermetic aluminium lids. Samples, under a nitrogen atmosphere, were equilibrated to − 10 °C before being ramped to 200 °C, at 10 °C min^–1^. Once samples had reached their max temperature, samples were cooled back down to − 10 °C and then re-heated to 200 °C, at 10 °C min^-1^. Samples were then re-cooled back down to − 50 °C, at 10 °C min^-1^. DSC was used to determine the glass-transition temperature (Tg), melting temperature (Tm), crystallisation temperature (Tc (melt)), and the cold-crystallisation temperature ((Tc (cold)) of the polymer.

### High-performance liquid chromatography (HPLC) analysis

The concentrations of glucose in culture supernatant were determined by a high-performance liquid chromatography (HPLC) system equipped with an RID10A refractive index detector (Shimadzu) at 50 °C. 20 µl of samples were injected into the column after the culture supernatant was filtered through Mini-UniPrep syringeless filter devices (Agilent). The glucose was analysed by an Aminex-87H column (Bio-Rad) at 40 °C. The samples in the column were eluted with 0.014 M H_2_SO_4_ at a flow rate of 0.55 ml/min under a pressure of 4.3 MPa.

## Supplementary Information

Additional file 1.

Additional file 2.

Additional file 3.

Additional file 4.

Additional file 5.

## Data Availability

No datasets were generated or analysed during the current study.
