# Supplementary material for: The link of carbon catabolite repression elements, small RNAs CrcY and CrcZ and polyhydroxyalkanoate metabolism in Pseudomonas putida KT2440
Source: Biotechnol Biofuels Bioprod. 2025 Oct 17;18:107. doi: 10.1186/s13068-025-02707-5 (PMC12533007; doi:10.1186/s13068-025-02707-5)
Supplement: Supplementary file 5 — Additional file 5. [file 13068_2025_2707_MOESM5_ESM.pdf]

Supplementary methods

CRISPR protocol details

The sgRNAs were designed using Synthego CRISPR Design Tool (https://design.synthego.com) to target the sequence of a specific *P. putida* KT2440 gene to be deleted. The SnapGene software (www.snapgene.com) was used to design the constructs and primers.

Proteomic protocol details

The cultures were grown as described above for PHA analysis. The following PHA-accumulating conditions were used: glucose as the carbon and energy substrate under nitrogen limitation (GNL), octanoate as the carbon and energy substrate with nitrogen limitation (ONL), and without limitation (ONF). The samples were at 21h, 18h, 15h respectively, what was predetermined to correspond to early stationary phase for each condition (Figure S1). Pellets were then washed twice with chilled phosphate buffer and stored at-80 °C until further analysis.

Sample preparation was carried out according to a previously published protocol [1]. In brief, frozen pellets were thawed on ice and resuspended in 8 M urea buffer, to a uniform protein concentration of 100 µg/µL. Firstly, cysteines were reduced using dithiothreitol (DTT) followed by alkylation with iodoacetamide (IAA). The samples containing DTT, IAA and urea were diluted using 50 mM ammonium Bicarbonate (NH_4HCO_3) before trypsinisation with trypsin singles proteomic grade (Sigma-Aldrich), ensuring a urea concentration of less than 2M. Digestion was carried out overnight at 37 °C. The digested samples were then acidified to pH 2.5 by the addition of 3 uL of formic acid and the peptides were then purified using ZipTip C18 columns (Merck Millipore, USA). The ZipTips were equilibrated with 20 µL acetonitrile and 20 µL 0.1 % (v/v) formic acid. To bind the peptides to the column, the peptide solution was aspirated 10 times. The bound peptides were washed with 0.1 % (v/v) formic acid and eluted in a clean LoBind Eppendorf with 40 µL 70 %(v/v) acetonitrile in 0.1 % (v/v) formic acid. After the concentrating step (1 h at 45 °C, Concentrator 5301; Eppendorf, Germany) the peptides were resuspended in 15 µL 0.1% (v/v) formic acid for the LC-MS/MS analysis. Proteomics samples were loaded onto individual EvoTips and run on a timsTOF Pro mass spectrometer (Bruker Daltonics, Bremen, Germany) coupled to the EvoSep One system (EvoSep BioSystems, Odense, Denmark). The peptides were separated on a reversed-phase C18 Endurance column (15cm x 100µm ID, C18, 3 µm) using the preset 30 SPD method. Mobile phases were 0.1% (v/v) formic acid in water (phase A) and 0.1% (v/v) formic acid in acetonitrile (phase B). The peptides were separated by an increasing gradient of mobile phase B for 44 minutes using a flow rate of 0.5 uL/min.

For Data Dependent Acquisition (DDA) the timsTOF Pro mass spectrometer was operated in positive ion polarity with TIMS (Trapped Ion Mobility Spectrometry) and PASEF (Parallel Accumulation Serial Fragmentation) modes enabled. The accumulation and ramp times for the TIMS were both set to 100 ms., with an ion mobility (1/k0) range from 0.6 to 1.6 Vs/cm. Spectra were recorded in the mass range from 100 to 1,700 m/z. The precursor (MS) Intensity Threshold was set to 2,500 and the precursor Target Intensity set to 20,000. Each PASEF cycle consisted of one MS ramp for precursor detection followed by 10 PASEF MS/MS ramps, with a total cycle time of 1.17 s.

Raw mass spectrometry data were processed using MaxQuant [2] (version 1.6.5.0), with protein identification performed against the *P. putida* KT2440 FASTA protein database (5527 entries), downloaded from UniProt on September 5, 2022. Potential contaminants, reverse sequence hits, and proteins identified solely based on modified peptides were filtered out using Perseus [3] software (version 1.6.5.0). Statistical analyses were conducted on the amica web platform [4] (version 3.0.0) using default parameters. Missing values were imputed using the platform's normal distribution-based method with a width of 0.8 and a downshift of 1.8. Differentially expressed proteins (DEPs) were identified using the "Analyze Differentially Abundant Proteins" function in Amica. Overexpression strains pCrcY and pCrcZ (*P. putida* KT2440 carrying plasmids pBT'T-crcY or pBT'T-crcZ, respectively) were compared to the control strain (*P. putida* KT2440 carrying the empty plasmid pBT'Tmcs) under three testing conditions: GNL, ONF, and ONL. All the fold changes reported in this proteomic study refer to $\log_2$-transformed fold changes between conditions, calculated based on Label-Free Quantification (LFQ) intensities, which reflect the relative abundance of each protein in the sample. DEPs were defined using a fold change threshold of 1 and p-value of less than 0.05 or proteins detected exclusively in overexpression strains or the control. Principal component analysis (PCA) was performed using the build-in function prcomp in R. The R package clusterProfiler was used for KEGG pathway enrichment analysis [5], and UpSetR was used to plot the UpSet graph [6].

Reference

1. Narancic T, Scollica E, Kenny ST, Gibbons H, Carr E, Brennan L, et al. Understanding the physiological roles of polyhydroxybutyrate (PHB) in Rhodospirillum rubrum S1 under aerobic chemoheterotrophic conditions. Applied Microbiology and Biotechnology. 2016;100:8901–12. https://doi.org/10.1007/s00253-016-7711-5.

2. Tyanova S, Temu T, Cox J. The MaxQuant computational platform for mass spectrometry-based shotgun proteomics. Nat Protoc. 2016;11:2301–19. https://doi.org/10.1038/nprot.2016.136.

3. Tyanova S, Temu T, Sinitcyn P, Carlson A, Hein MY, Geiger T, et al. The Perseus computational platform for comprehensive analysis of (prote)omics data. Nat Methods. 2016;13:731–40. https://doi.org/10.1038/nmeth.3901.

4. Didusch S, Madern M, Hartl M, Baccarini M. amica: an interactive and user-friendly web-platform for the analysis of proteomics data. BMC Genomics. 2022;23:817. https://doi.org/10.1186/s12864-022-09058-7.

5. Yu G, Wang L-G, Han Y, He Q-Y. clusterProfiler: an R Package for Comparing Biological Themes Among Gene Clusters. OMICS: A Journal of Integrative Biology. 2012;16:284–7. https://doi.org/10.1089/omi.2011.0118.

6. Conway JR, Lex A, Gehlenborg N. UpSetR: an R package for the visualization of intersecting sets and their properties. Bioinformatics. 2017;33:2938–40. https://doi.org/10.1093/bioinformatics/btx364.

Supplementary Tables:

File name: TS1 strains and plasmid list.xlsx

Title of data: Table S1 The strains, plasmids and oligonucleotides used in this study.

Legend : (A) Strains created and use in this study; (B) Plasmids used in this study; (C) Oligonucleotides used in this study.

File name: TS2 transporters summary.xlsx

Title of data: Table S2 Up-regulated transporters in both overexpression strains

Legend: Transporter proteins that were significantly up-regulated in both *pCrcY* and *pCrcZ* compared to the control strain under GNL, ONL, and ONF conditions ($P < 0.05$ and logFC > 1). The log fold change (*logFC*) of *pCrcY*/*pCrcZ* vs. the control strain is shown in the cells.

File name: TS3 proteomic raw data.xlsx

Title of data: Table S3 Proteomic raw data and statistical analysis results of pCrcY/Z and control strains

Table S4 : PHA content (%CDW) in wild-type, mutant, and plasmid-carried *P. putida* KT2440 strains grown under GNL, ONF and ONL conditions.

The wild-type KT2440 strain, single-deletion strains (KT Δhfq and KT Δcrc), the double-deletion strain (KT ΔΔhfq crc), and their corresponding in trans overexpression strains carrying either *pBT'T-crcY* (pCrcY) or *pBT'T-crcZ* (pCrcZ) were cultivated for 48 hours in MSM supplemented with glucose (G) and octanoate (O). The experiments were conducted under two nitrogen conditions: nitrogen-full (NF) and nitrogen-limited (NL).

	Strains	PHA(%CDW)	Strains	PHA(%CDW)	Strains	PHA(%CDW)
GNL	WT	39.71 ± 0.51	Con	19.71 ± 2.63	Con	16.02 ± 0.94
	Δ*hfq*	1.20 ± 0.09	ΔC-pE	24.58 ± 0.52	ΔH-pE	0.76 ± 0.16
	Δ*crc*	20.27 ± 2.37	ΔC-pCrcY	21.42 ± 1.09	ΔH-pCrcY	0.74 ± 0.06
	ΔΔ*hfq crc*	1.38 ± 0.09	ΔC-pCrcZ	21.55 ± 1.86	ΔH-pCrcZ	0.79 ± 0.08
ONF	WT	19.26 ± 1.07	Con	14.65 ± 0.72	Con	13.22 ± 1.06
	Δ*hfq*	13.20 ± 0.77	ΔC-pE	15.61 ± 0.36	ΔH-pE	14.50 ± 0.79
	Δ*crc*	17.60 ± 1.09	ΔC-pCrcY	12.01 ± 2.40	ΔH-pCrcY	9.18 ± 0.70
	ΔΔ*hfq crc*	16.15 ± 0.88	ΔC-pCrcZ	13.22 ± 0.90	ΔH-pCrcZ	11.75 ± 1.30
ONL	WT	62.17 ± 2.56	Con	24.84 ± 1.36	Con	23.88 ± 0.65
	Δ*hfq*	65.04 ± 0.94	ΔC-pE	21.93 ± 1.48	ΔH-pE	28.56 ± 3.03
	Δ*crc*	61.92 ± 2.56	ΔC-pCrcY	25.68 ± 0.55	ΔH-pCrcY	30.56 ± 0.85
	ΔΔ*hfq crc*	68.30 ± 0.99	ΔC-pCrcZ	25.30 ± 4.61	ΔH-pCrcZ	31.59 ± 4.42

File name: TS5 DSC analysis raw data.xlsx

Table S5. Differential scanning calorimetry (DSC) raw data of mcl-PHA samples, including first heating, cooling, and second heating cycles.

Supplementary figures:

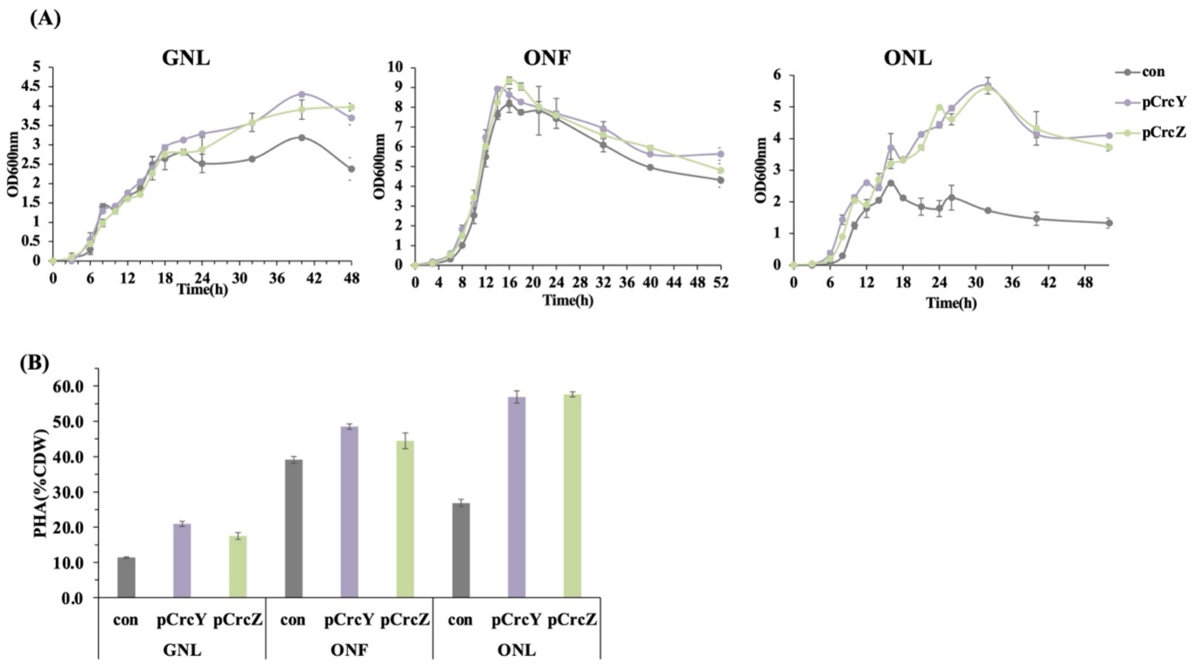

Fig S1: Growth curve and PHA level of pCrcY and pCrcZ strains in proteomic tested condition

(A) The small RNA CrcY and CrcZ overexpression (purple line representing pCrcY and green line representing pCrcZ) strains and the KT2440 strain transformed with an empty pBT'Tmcs vector (grey line representing con) were cultivated for 48 hours in defined medium MSM supplemented with octanoate or glucose, with nitrogen conditions as nitrogen-full (NF) or nitrogen-limited (NL). Growth was followed by measuring turbidity at 600 nm. (B) The PHA accumulation levels in the pCrcY and pCrcZ and the control strains at the proteomic sampling time points.

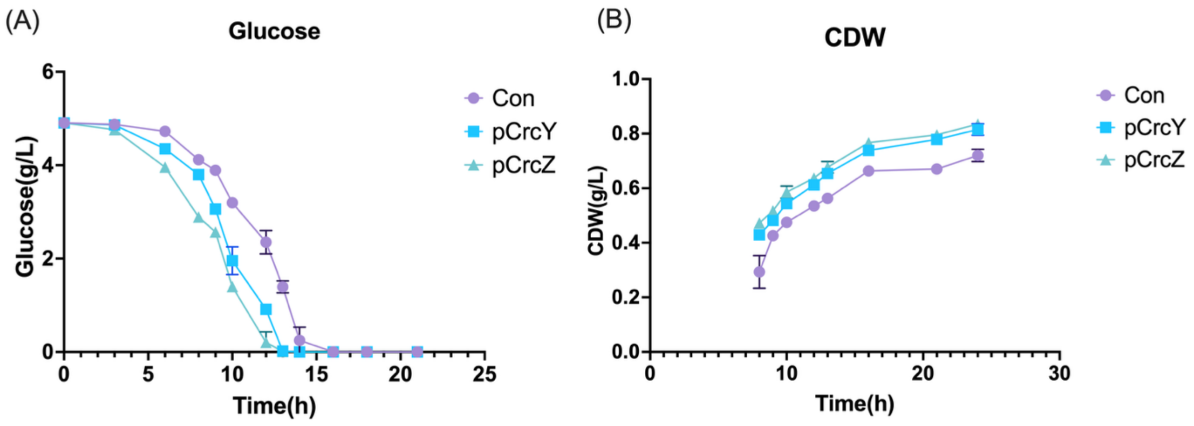

Figure S2. Cell dry weight (CDW) and glucose consumption of pCrcY and pCrcZ strains under GNL conditions. Strains overexpressing small RNAs CrcY (blue) and CrcZ (green), along with the control strain carrying the empty pBT'Tmcs vector (purple), were

cultivated in defined MSM medium supplemented with glucose under nitrogen-limited (GNL) conditions for 24 hours. (A) Glucose concentrations in the culture supernatant. (B) Cell dry weight measurements at each sampling time point.

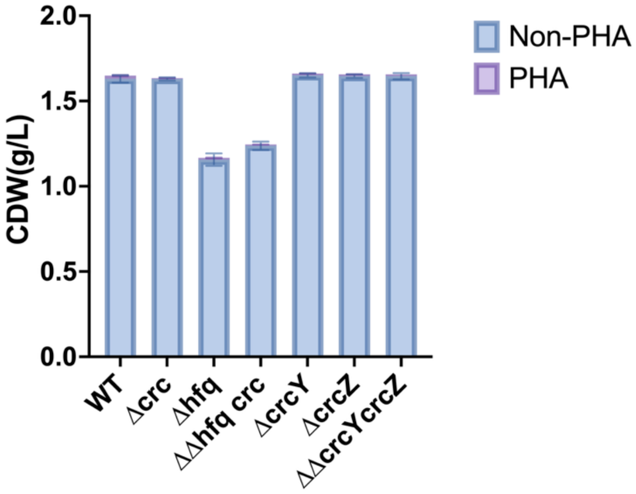

Fig S3: Biomass and PHA accumulation of CCR components deletion strains under GNF conditionKT Δ*hfq* and KT Δ*crc*, the double-deletion strain KT ΔΔ*hfq crc*, KT Δ*crcY* and KT Δ*crcZ*, the double-deletion strain KT ΔΔ*crcY crcZ*, and the wild-type KT2440 were cultivated in MSM nitrogen-full media supplemented with glucose for 48 hours.

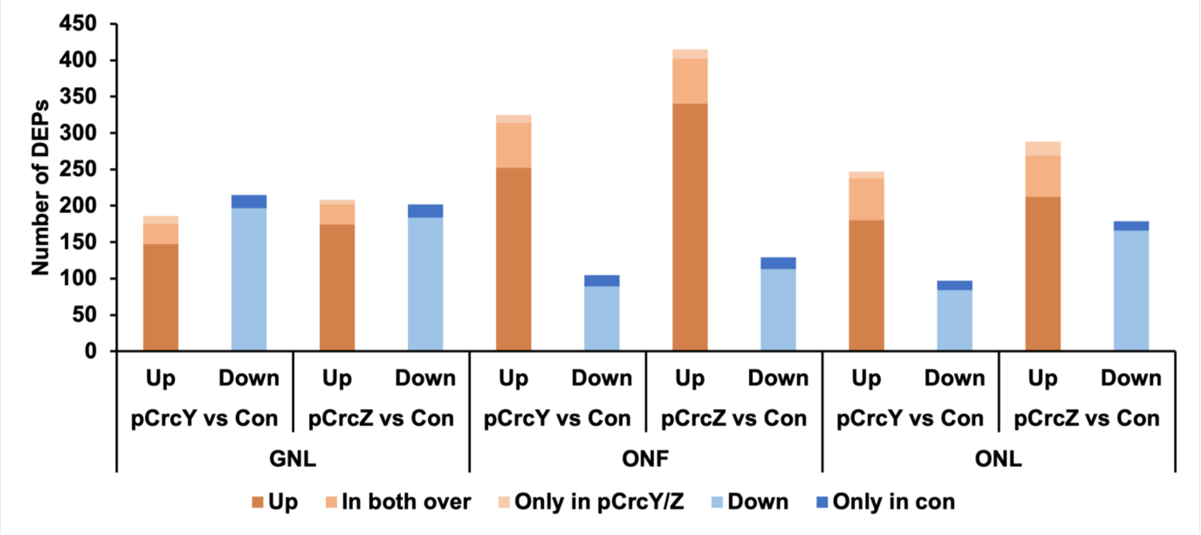

Fig S4: The number of differentially expressed protein in pCrcY or pCrcZ compared to the control.

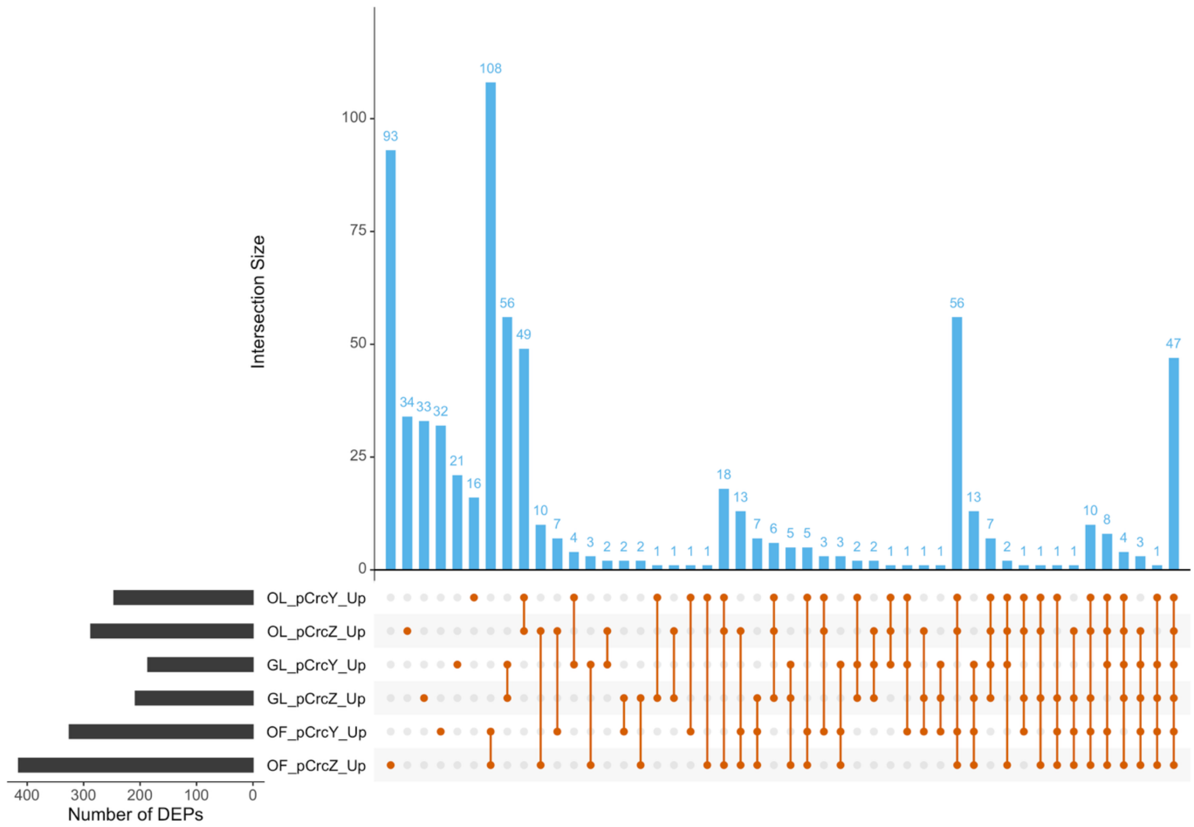

Fig S5: The Upset Plot of proteins with higher abundance in pCrcY and pCrcZ strains compared to the control.

The plot shows the number of **upregulated proteins** across different conditions. Horizontal bars on the left represent the total number of upregulated proteins in each strain and condition (GNL, ONF, and ONL). Vertical bars indicate the size of intersecting sets, highlighting proteins that are shared or unique between conditions and strains.

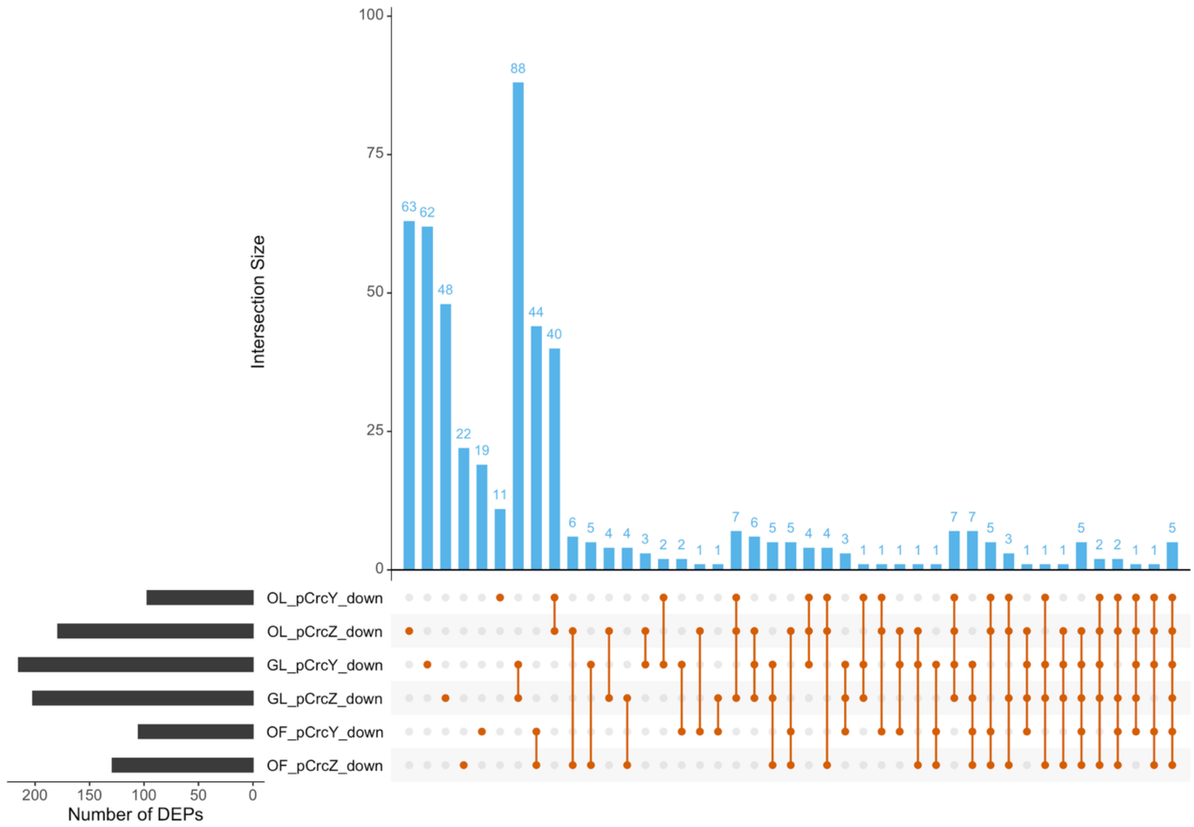

Fig S6: The Upset Plot of proteins with lower abundance in pCrcY and pCrcZ strains compared to the control.

The plot shows the number of **proteins with decreased abundance** across different conditions. Horizontal bars on the left represent the total number of upregulated proteins in each strain and condition (GNL, ONF, and ONL). Vertical bars indicate the size of intersecting sets, highlighting proteins that are shared or unique between conditions and strains.

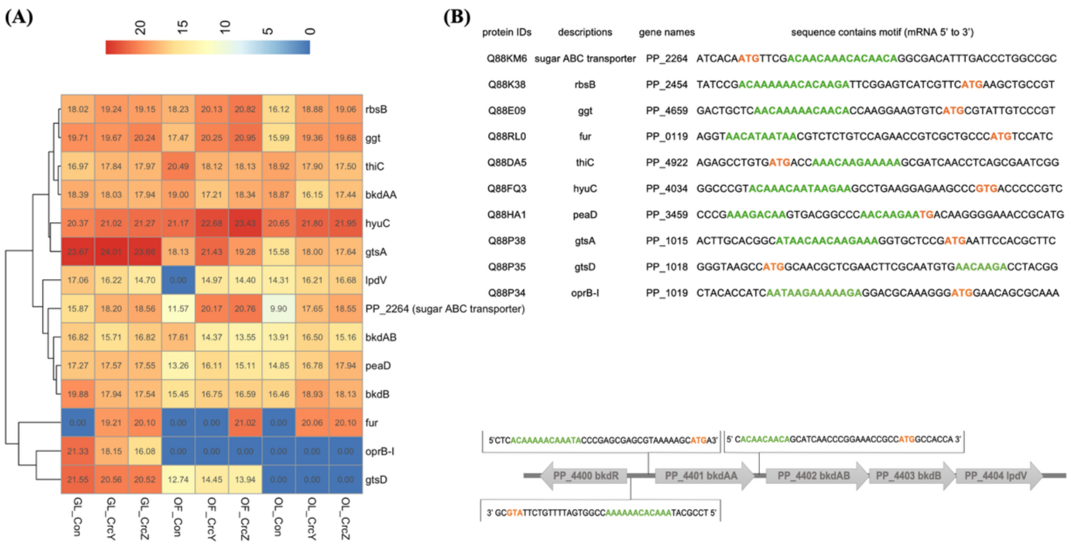

Fig S7: Possible targets for Hfq/Crc regulation in *P. putida* KT2440

(A)The heatmap of expression level of potential target genes in pCrcY and pCrcZ and control stains; (B) the sequences of potential target genes, the start codons are colored in orange and the CA motifs are colored in green.

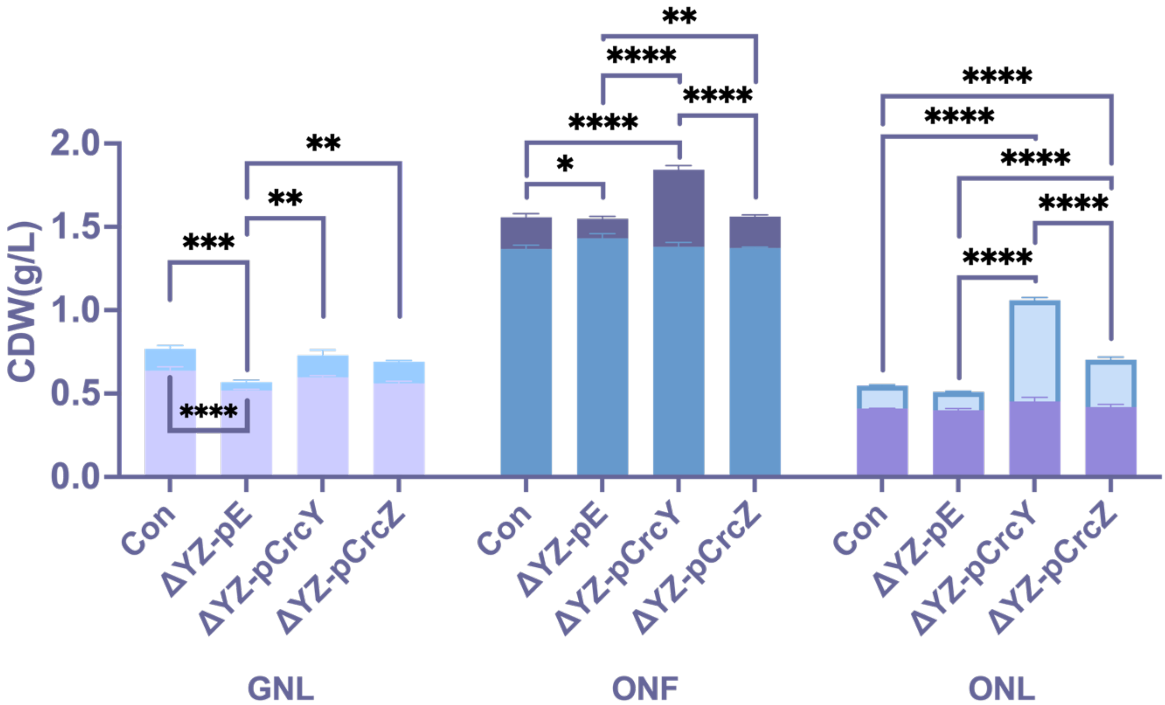

Fig S8 Biomass and PHA accumulation in P. putida KT2440 CrcY and CrcZ complementation strains.

CrcY or CrcZ was expressed from the pBT'Tmcs vector in ΔΔ*crcYcrcZ* (ΔYZ-pCrcY or ΔYZ-pCrcZ), or an empty pBT'Tmcs vector was used in ΔΔ*crcYcrcZ* (ΔYZ-pE). KT2440 wild-type carrying the pBT'T-empty plasmid served as the control (Con). The strains were cultivated

for 48 hours in MSM supplemented with glucose (G) and octanoate (O), with nitrogen conditions indicated as nitrogen-full (NF) and nitrogen-limited (NL). PHA amounts are represented by upper bars, while biomass (excluding PHA) is shown by bottom bars. Asterisks indicate significant differences between the samples and the control, or the comparison indicated in brackets: * P $\leqslant$ 0.05, ** P $\leqslant$ 0.01, *** P $\leqslant$ 0.001, **** P $\leqslant$ 0.0001.

Description:

To further confirm that the observed effect on PHA levels was due to the deletion of CrcY and CrcZ, pBT'T-empty, pBT'T-crcY, and pBT'T-crcZ constructs were introduced into KT2440 WT and ΔΔ*crcYcrcZ*. Under GNL conditions, KT ΔΔ*crcYcrcZ_pE* showed a 18.6% decrease in the non-PHA portion of biomass and a 61.5% decrease in PHA content compared to the control. Under ONF conditions, the PHA content was only 7.6% CDW, which is 37.7% lower than the control (12.2% CDW) grown under the same conditions. No statistically significant differences were observed under ONL conditions for either the residual biomass or PHA levels. However, the reduction in PHA levels was alleviated by the complemented expression of sRNAs from pBT'T_CrcY or pBT'T_CrcZ. Under GNL PHA accumulating conditions, the overexpression of either CrcY or CrcZ in KT ΔΔ*crcYcrcZ* led to the restoration of PHA level observed in the control (Fig. 6). Under ONF conditions, overexpression of CrcY or CrcZ resulted in varying levels of PHA accumulation. KT ΔΔ*crcYcrcZ_pCrcY* exhibited a 2.4-fold increase in PHA levels compared to the control, while KT ΔΔ*crcYcrcZ_pCrcZ* showed PHA levels comparable to the control (Fig. 2B). Under ONL conditions, both pCrcY and pCrcZ led to increased PHA accumulation in KT ΔΔ*crcYcrcZ* compared to the control, with PHA comprising 57.2% CDW and 40.4% CDW, respectively.

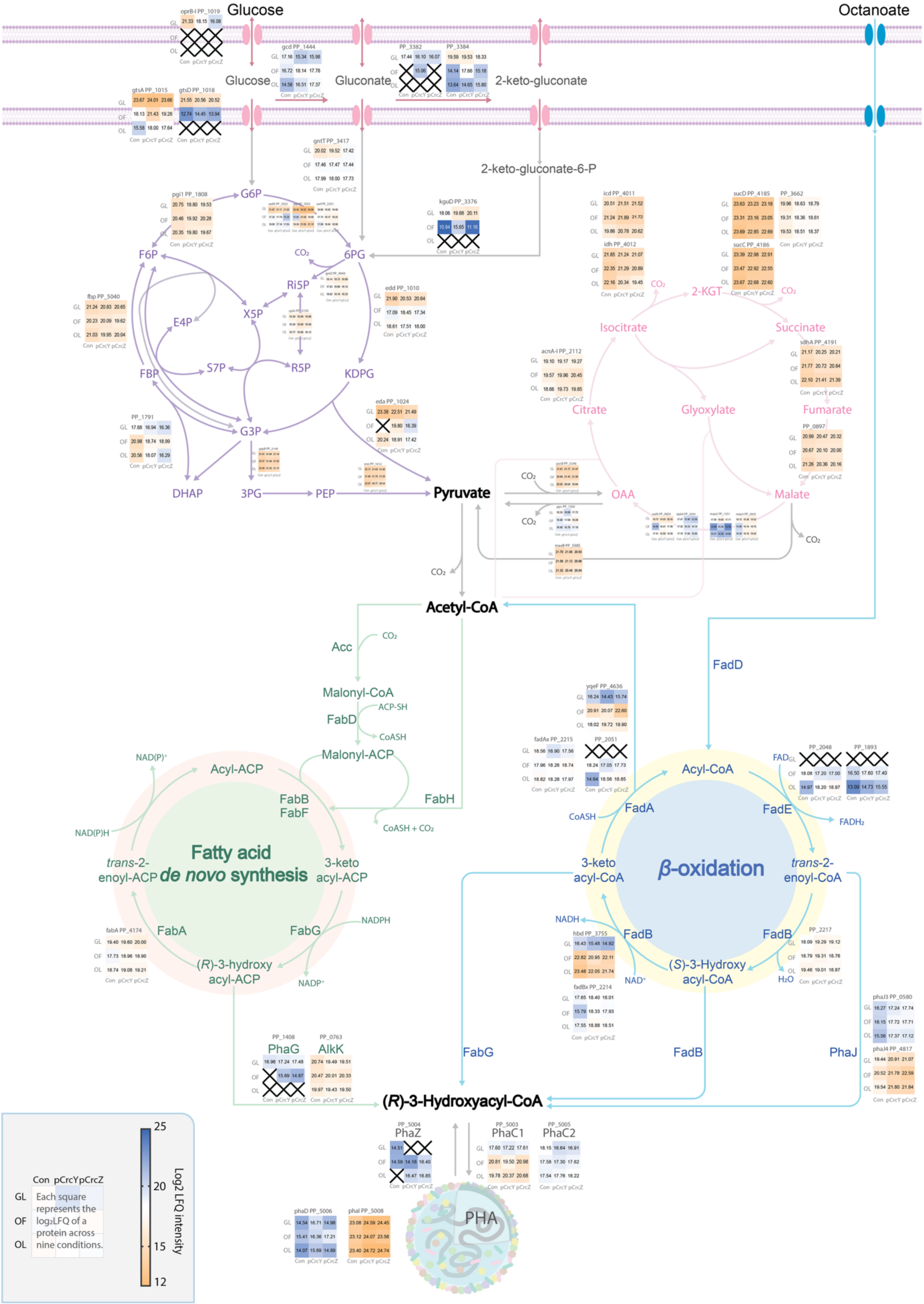

Fig S9: Differentially expressed proteins (DEPs) involved in the glucose and PHA metabolism.

Each square represents the log_2LFQ intensity of a protein under three different culture

conditions: glucose with nitrogen limitation (GL), octanoate with full nitrogen (OF), and octanoate with nitrogen limitation (OL). Data are shown for three strains: the control strain carrying the empty pBT'T plasmid (Con), and strains overexpressing either CrcY or CrcZ (pCrcY or pCrcZ). Blue tones indicate lower protein abundance, while orange tones indicate higher abundance.

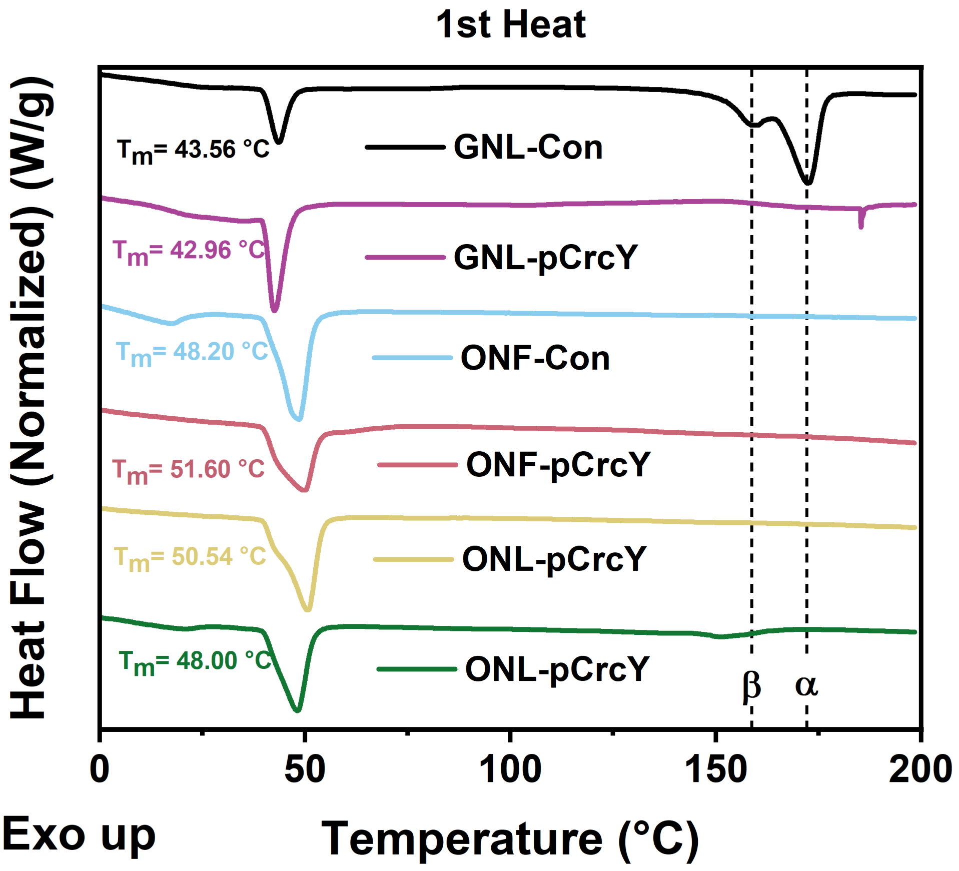

Fig S10: Differential scanning calorimetry (DSC) thermograms of PHA samples produced by *P. putida* strains under different conditions.

DSC analysis was performed on PHA samples obtained from control (Con) and *crcY* overexpression (pCrcY) strains cultivated under glucose–nitrogen-limited (GNL), octanoate–nitrogen-full (ONF), and octanoate–nitrogen-limited (ONL) conditions. The melting temperature (Tm) values of the major endothermic transitions are indicated for each sample. GNL-control sample exhibited additional high-temperature endothermic peaks at approximately 153 °C (β-phase) and 168 °C (α-phase), corresponding to distinct crystalline polymorphs of PHA.
